# Carboxylate-Ligand-Based Coordination Polymers and Metal Complexes: From Structural Diversity and Supramolecular Descriptors to Function-Oriented Design

**DOI:** 10.3390/molecules31173059

**Published:** 2026-08-31

**Authors:** Xiangjun Kong, Xia Wang, Xishi Tai

**Affiliations:** School of Chemistry & Chemical Engineering and Environmental Engineering, Weifang University, Weifang 261061, China

**Keywords:** carboxylate ligands, coordination polymers, metal complexes, supramolecular descriptors, structure–function relationships, descriptor-guided function-oriented design

## Abstract

Carboxylate-ligand-based coordination polymers and metal complexes form structurally adaptable crystalline systems, spanning discrete complexes, one-dimensional chains, two-dimensional layers, three-dimensional frameworks, and MOF-like architectures. This adaptability arises from diverse carboxylate binding modes and metal coordination preferences; auxiliary N/O donors are included only when carboxylate coordination remains central. However, the increasing availability of structural and electronic descriptors has not always been matched by equally rigorous validation of structure–function relationships. Hirshfeld surface analysis, energy-framework analysis, density functional theory, adsorption simulation, molecular docking, and machine learning are descriptor-generating methods; neither a method nor its output alone establishes descriptor-guided design. This review critically examines carboxylate-based coordination systems from the perspective of descriptor-guided function-oriented design. We discuss ligand-level regulation, metal-center effects, dimensional evolution, and what supramolecular, electronic, adsorption-related, and biological descriptors can and cannot prove. Functional studies covering luminescence and sensing; catalysis, adsorption, and small-molecule transformations; and bioactivity are evaluated by evidence strength. A claim-centered Descriptor-to-Function Evidence Ladder distinguishes structure reporting, descriptive descriptor use, post hoc association, controlled comparative trends, mechanism-supported validation, and prospective experimental confirmation. It evaluates a specific descriptor–function relationship rather than overall paper quality. Future progress should rely on standardized reporting, comparative structural series, function-specific validation, and transferable descriptor–function relationships.

## 1. Introduction

Metal complexes and coordination polymers based on carboxylate ligands constitute a major and structurally diverse family of coordination compounds at the interface of coordination chemistry, crystal engineering, and functional materials science. The popularity of the carboxylate family is due to its inherent chemical property: a single carboxylate motif can serve as a monodentate donor, a bidentate chelator, a syn–syn or syn–anti bridge, or a multinuclear linker, giving rise to discrete metal complexes, coordination chains, and layered and three-dimensional frameworks [1,2]. The structural versatility of carboxylate ligands makes such compounds not only attractive for crystal engineering purposes but also interesting from the point of view of supramolecular function-oriented research, since they allow exploring the role of ligand structure, metal–ligand coordination, and resulting supramolecular environments in defining material functions.

In porous coordination polymers and metal–organic frameworks, metal–carboxylate motifs are central building blocks in reticular synthesis [3,4] and SBU/topology-based framework design [5,6,7,8]. In molecular and low-dimensional systems, the same carboxylate chemistry enables mononuclear, dinuclear, polynuclear, and chain motifs that can be defined crystallographically. Metal positions, ligand conformations, carboxylate coordination modes, hydrogen bonds, aromatic contacts, and crystal voids can therefore provide chemically meaningful structural descriptors. Additional N-donor ligands such as 1,10-phenanthroline and 2,2′-bipyridine commonly act as chelating or capping co-ligands, whereas 4,4′-bipyridine and selected imidazole or triazole derivatives can bridge metal centers; these roles can terminate or extend a carboxylate-based coordination network, alter packing, or modify the local electronic environment [9]. In this review, such N-containing ligands are included only when a carboxylate ligand anchors or materially participates in the same metal–ligand architecture. Coordination systems based exclusively on N-donor ligands are outside the scope. The main question is how these structural variables can be translated into descriptors that are not merely reported but tested for their ability to explain or predict function.

Against this structural background, the scope of functions of carboxylate-based coordination compounds has significantly grown [10]. Metal–organic frameworks and coordination polymers showing luminescent properties have been used for ion detection, solvent recognition, small-molecule sensing, and guest-responsive emission [11,12]. Emission behavior can involve ligand-centered or metal-centered excited states, ligand-to-metal or metal-to-ligand charge transfer, and lanthanide sensitization, while framework rigidity and host–guest interactions may modulate emission intensity and lifetime [13]. At the same time, other directions for applications have emerged. Porous metal–carboxylate frameworks have been widely investigated as heterogeneous catalysts, adsorbents, and separation media because of their tunable metal nodes, pore environments, and functional groups [14]. Crystallographically characterized nonporous metal–carboxylate compounds have also been investigated in catalysis and small-molecule transformations, for which reactivity may arise from Lewis-acidic or redox-active metal centers, substrate-accessible coordination sites, or ligand-mediated electronic effects [15]. Biological and biomedical studies have also expanded, including studies of transition-metal carboxylate complexes evaluated for cytotoxicity, DNA or protein interactions, and antibacterial activity [16,17]. The expansion of functional scope was paralleled by the rapid growth of descriptor-generating approaches. Hirshfeld surface analysis and energy-framework analysis are used to characterize intermolecular contacts and crystal packing; their outputs include mapped surface features, contact fractions, and model-dependent interaction energies. DFT and TD-DFT generate electronic and excited-state outputs; GCMC simulation generates adsorption-related predictions; molecular docking generates poses and scores; and machine-learning methods generate learned representations and predictions [18,19]. These approaches are valuable, but their outputs must be separated from evidence that validates a structure–function relation. For instance, a Hirshfeld fingerprint plot may show high contributions of O···H or H···H contacts in the crystal packing of some compounds, but it is not evidence that a compound quenches fluorescence through intermolecular interactions. Electropositive and electronegative regions may be identified through electrostatic potential maps, but this does not mean that they are responsible for selective adsorption or biomolecular binding. Differences in HOMO–LUMO gaps between two compounds may suggest electronic trends, but they cannot explain catalytic rate, origin of emission, or cytotoxic mechanism. The key issue here is not the lack of descriptors but the insufficient link between descriptors and function.

Existing reviews and perspectives on coordination polymers, MOFs, and carboxylate systems provide important but differently framed backgrounds. Some focus on terminology, net/topological description, or reticular design [20,21,22], whereas others extend reticular concepts toward biological systems [23]. Recent specialized reviews have examined Cd(II)- and Zn(II)-carboxylate coordination polymers and carboxylate-based MOF/coordination-polymer glasses [24,25,26]. These reviews provide focused discussions of particular compound classes or applications, but they generally do not integrate crystal-structure description, supramolecular and electronic descriptors, function-specific validation, and explicit evaluation of evidence strength within a single framework. This distinction is important because the use of a descriptor does not itself constitute descriptor-guided function-oriented design. Depending on its use, a descriptor may remain descriptive, support post hoc or comparative correlations, contribute to mechanism-supported validation, or be used prospectively for prediction. In many carboxylate coordination studies, descriptors are calculated/visualized after structure determination and used to interpret the observed function. More advanced studies generate structural series, test descriptor-derived hypotheses, or employ descriptors prospectively to predict a function before experiments.

To address this gap, this review evaluates carboxylate-ligand-based coordination polymers and metal complexes in the context of descriptor-guided function-oriented design. It is not intended to be an exhaustive overview of all carboxylate-containing crystal structures or an application-by-application survey of luminescence and sensing, catalysis, adsorption, small-molecule transformations, and biological activity. Instead, it focuses on how ligand design, metal-center selection, structural dimensionality, supramolecular interactions, and structural and electronic descriptors are linked to functional performance at different levels of evidence.

The general thesis of this review is that the community of researchers dealing with metal complexes and coordination polymers should move from simple structure reporting to structure–function evidence and eventually to prospective prediction. The guiding logic is as follows: carboxylate-ligand design and metal-center selection, under defined synthetic conditions → coordination mode and local coordination environment → structural dimensionality and supramolecular organization → descriptor extraction → functional evaluation → evidence grading → feedback to design. The key question is not only whether a compound is novel or functional, but whether the reported descriptors support the claimed function and how strong the corresponding descriptor–function evidence is.

**Figure 1:**
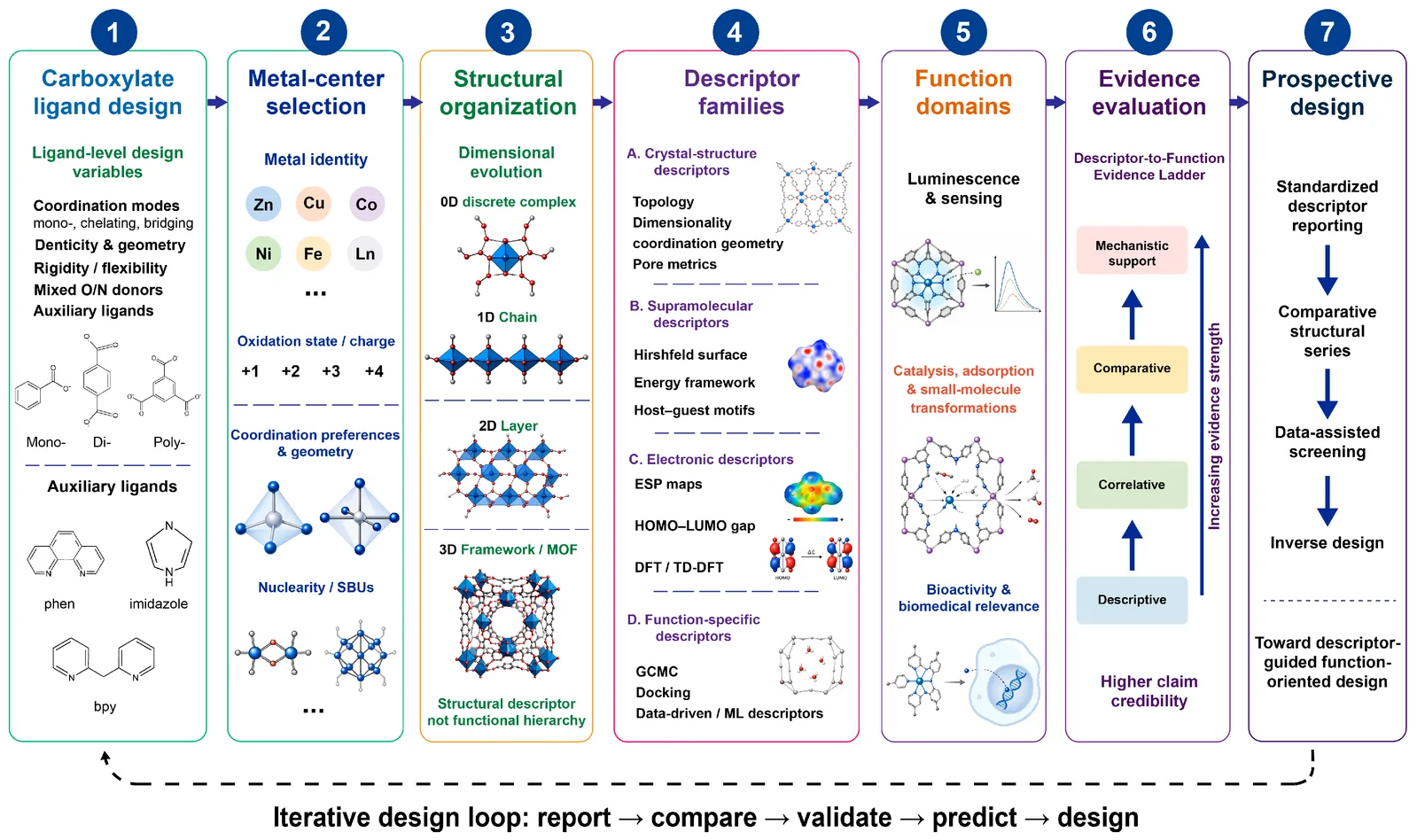
Overall framework of this review. Carboxylate-ligand design and metal-center selection influence structural organization, from which structural, supramolecular, electronic, and function-specific descriptors are extracted. Functional claims are then assessed using the Descriptor-to-Function Evidence Ladder, with iterative feedback supporting increasingly predictive design. Solid arrows show conceptual progression; upward arrows indicate increasing evidentiary support; the dashed re-turn arrow denotes design feedback.

To answer this question, the review proposes a Descriptor-to-Function Evidence Ladder. Its intended contribution is not another list of known cautions about individual methods; rather, it supplies a claim-centered decision logic for stating what a specific descriptor–function inference is supported to establish. The ladder classifies claims from Level 0, where only structural identity is established, to Level 5, where a descriptor-based prediction is specified prospectively and then confirmed experimentally. It separates method from descriptor output, distinguishes co-reporting from controlled comparison and mechanism-supporting validation, and records why a claim is capped at a given level. The general logic is shown in Figure 1. Carboxylate-ligand design and metal-center selection jointly influence, rather than uniquely determine, local coordination and structural dimensionality. These structural features provide a basis for extracting supramolecular, electronic, adsorption-related, and biological descriptors; functional claims are then evaluated according to the evidence connecting a defined descriptor to performance.

Following this framework, Section 2 discusses carboxylate ligands as structural regulators, emphasizing coordination modes, ligand geometry, mixed O/N-donor systems, and auxiliary-ligand strategies. Section 3 analyzes how metal centers influence local coordination, nuclearity, secondary building units, and dimensional evolution from discrete complexes to three-dimensional frameworks. Section 4 provides the evidence-evaluation core of the review by distinguishing descriptor-generating methods from their outputs, including Hirshfeld-surface contact descriptors, energy-framework interaction energies, molecular and periodic DFT-derived electronic descriptors, and function-specific outputs; it then addresses over-interpretation and the Descriptor-to-Function Evidence Ladder. Section 5 then applies this framework to luminescence and sensing; catalysis, adsorption, and small-molecule transformations; and bioactivity. Section 6 provides the forward-looking design core by proposing a roadmap for standardized descriptor reporting, comparative structural series, function-specific validation, data-assisted screening, and inverse design, before Section 7 summarizes the conceptual message and future directions of the field.

## 2. Carboxylate Ligands as Structural Regulators

In carboxylate-based coordination systems, the ligand is a primary structural variable. The binding mode of a carboxylate group is not fixed, since a carboxylate ligand can coordinate in terminal, chelating, bridging, and chelating–bridging modes. Along with ligand denticity, rigidity, flexibility, donor-site arrangement, aromatic surface, hydrogen bonding ability, and auxiliary donor sites, these ligand binding modes influence the local coordination sphere, dimensional extension, supramolecular packing, and possible functional behavior [27].

Due to this flexibility, it is also harder to predict carboxylate-based framework structures. The same ligand can give rise to a discrete complex, chain, layer, or framework depending on pH, solvent, counter-ion, auxiliary ligand, temperature, concentration, and type of metal center [28,29,30]. Hence, carboxylate ligands will not be analyzed here as a catalogue of building blocks, but rather as ligand-level variables, from which structural descriptors can be generated and utilized in further function-related studies.

### 2.1. Coordination Mode as the First Ligand-Level Descriptor

Coordination mode is a direct ligand-level descriptor. Usually, monodentate binding occurs in a terminal environment, chelation can stabilize local metal coordination spheres, and bridging coordination connects metal centers, thus influencing metal-to-metal distance and propagation direction. Chelating–bridging and higher-order bridging modes can increase connectivity and support polynuclear units, chains, layers, and even frameworks.

This ligand-level descriptor influences metal-center accessibility, network extension, framework rigidity, pore formation, and supramolecular packing. Still, coordination mode does not directly define functional properties. For example, a bridging carboxylate ligand may help in framework formation, yet it is not sufficient to claim good adsorption performance. Similarly, a chelating ligand may create a stable complex, yet it does not by itself explain any catalytic or biological activity. Further descriptor extraction and validation are required for that purpose.

### 2.2. Ligand Connectivity, Geometry, and Conformational Control

Ligand connectivity and geometry affect the extent to which coordination can occur. Recent Cu(II) and Ca(II) coordination polymers based on triazolyl dicarboxylates illustrate how ligand connectivity and donor-site geometry can direct extended assembly [31,32]. Monocarboxylates often form discrete or low-dimensional assemblies but can also support extended structures through bridging coordination, while di- and polycarboxylates can connect multiple metal centers and form extended networks [33,34,35]. While rigid aromatic ligands can provide predictable coordination vectors and robust frameworks, flexible and semi-rigid ligands can increase conformational variability, polymorphism, framework dynamics, and structural unpredictability [36,37,38,39]. Topological disorder provides an additional, distinct route to elastic stability [40].

Carboxylate-based frameworks illustrate how ligand size, angularity, and flexibility, together with defect chemistry, affect connectivity, pore environment, and framework dynamics. Rigid polycarboxylate linkers and highly connected metal–carboxylate clusters allow formation of robust reticular structures [41,42], while aliphatic, Sn(II), glass-like [43,44], and defect-tailored coordination polymers allow expansion of the design space beyond ideal rigid-linker frameworks [37,43,45].

### 2.3. Mixed-Donor and Auxiliary-Ligand Regulation

Phenoxyacetate, pyridyl-carboxylate, isonicotinate, and similar mixed-donor ligands bridge primary coordination with supramolecular and electronic descriptors. Phenoxyacetate ligands feature a carboxylate anchor, aromatic surface, and a flexible ether linker, while pyridyl-carboxylates provide both O- and N-donor sites that can complete coordination spheres or extend coordination networks [46]. These properties may affect framework emission properties, guest recognition, catalytic microenvironment, or bioactivity, yet their functional roles should be tested rather than assumed.

These systems provide a useful testing platform for ligand-level design since they allow generating descriptors at several different levels: metal coordination, connectivity, hydrogen bonding, aromatic packing, and electron distribution. Coordination polymers of sodium complexes [47,48], Cu(II) complexes, heterometallic Cu–lanthanide coordination polymers, and supramolecular polyhedra based on nanosized carboxylate ligands illustrate how ligand geometry can be translated into packing and a functional environment via primary coordination and secondary interactions [49,50,51].

N-donor functionality is included only in mixed-donor carboxylates [52] or as an auxiliary co-ligand in carboxylate-based systems [53,54,55]. Purely N-donor coordination polymers are excluded; N,O-donor Schiff-base systems are only methodological comparators [56]. Inclusion does not attribute function uniquely to the carboxylate; donor-set roles require comparison or claim-specific validation.

### 2.4. Ligand-Level Principles for Descriptor-Guided Design

Ligand-level principles can therefore be summarized as follows: coordination mode defines local connectivity; ligand connectivity and donor-site arrangement influence the extent of coordination; ligand rigidity and flexibility balance predictability and variability; the presence of aromatic groups and heteroatoms affects packing and electron distribution; auxiliary donor ligands allow tuning of dimensionality and metal site environment. These variables are aligned with the recent trends in coordination chemistry and porous materials chemistry [57], where control of local coordination and framework dynamics become increasingly important [58].

**Figure 2:**
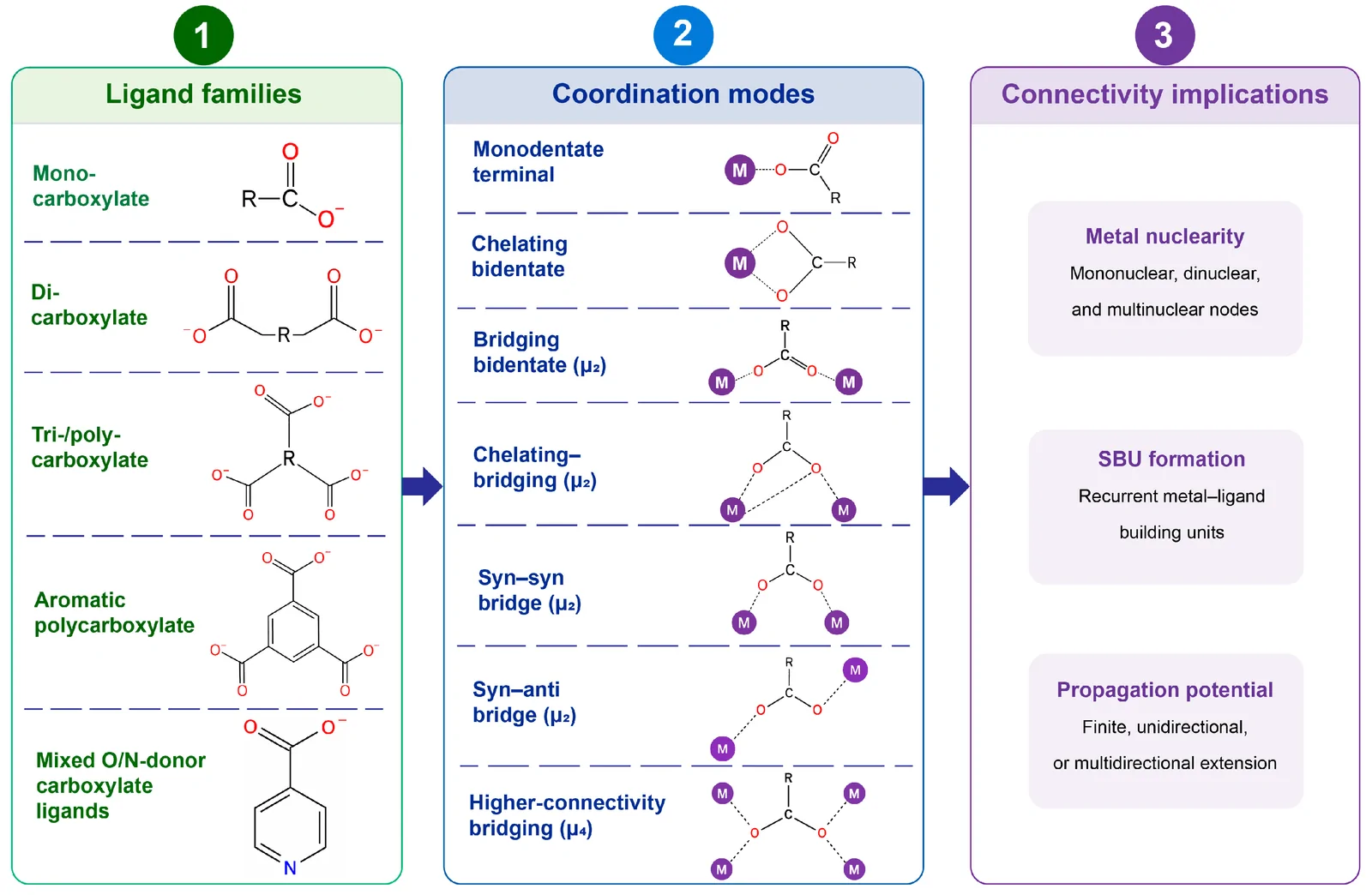
Representative carboxylate ligand families, coordination modes, and connectivity implications. Monocarboxylate, dicarboxylate, tri-/polycarboxylate, aromatic polycarboxylate, and mixed O/N-donor carboxylate ligands can adopt terminal, chelating, and bridging coordination modes, including chelating–bridging, syn–syn, syn–anti, and higher-connectivity bridging arrangements. These local binding patterns influence metal nuclearity, the formation of recurrent metal–ligand secondary building units (SBUs), and the potential for finite, unidirectional, or multidirectional structural propagation. The coordination motifs are schematic representations intended to illustrate local connectivity rather than exact crystallographic geometries.

Ligand design alone does not guarantee functionality. Instead, ligand identity and local coordination modes define the initial connectivity landscape, including metal nuclearity, secondary building unit formation, and the potential for structural propagation. Descriptor-guided function-oriented design, therefore, requires evidence linking these structural consequences to measurable functional performance. This transition from ligand-level variables to descriptor–function relationships provides the conceptual bridge to the descriptor-focused framework developed in Section 4. Representative carboxylate ligand families, coordination modes, and their connectivity implications are summarized in Figure 2 and Table 1.

## 3. Metal-Center Effects and Dimensional Evolution

Metal centers comprise the second major design parameter of carboxylate coordination chemistry. They strongly influence coordination number, geometry, nuclearity, Lewis acidity, redox activity, photophysical behavior, and framework connectivity; therefore, the same carboxylate ligand may yield different structural and functional outcomes depending on the chosen metal [29]. In the current review, metal centers are regarded not merely as a classification parameter, but rather as the sources of structural and electronic descriptors, which have to be related to function via evidence.

### 3.1. Alkali- and Alkaline-Earth-Metal Centers

Alkali- and alkaline-earth-metal ions, including Na(I), K(I), Mg(II), Ca(II), Sr(II), and Ba(II), tend to be oxygenophilic and adopt relatively high or variable coordination numbers, reflecting their ionic radii, charge densities, and affinity for hard O donors. These features can favor the formation of extended metal–carboxylate networks, whereas accessible Lewis-acidic sites depend on metal identity and coordination environment. The recent examples of Ca(II) and Ba(II) coordination polymers from 3-(3-carboxyphenyl) isonicotinic acid highlight how crystallographic descriptors, DFT-derived electronic outputs, and benzyl alcohol oxidation tests can be used in alkaline earth metal systems [59,60]. Still, these systems require the same core validation principles as for transition metal catalysts or porous frameworks: controls, recycling, and structure retention for catalysis claims and pore accessibility, isotherms, and regeneration and competitive conditions for adsorption studies.

### 3.2. Transition-Metal Centers

Transition-metal ions offer a wider variety of geometries and electronic states. For example, coordination compounds of Co(II), Ni(II), Cu(II), Mn(II), Fe(III), Zn(II), and Cd(II) may occur as discrete complexes, chains, layers, clusters, or frameworks. Partially occupied d shells provide redox, magnetic, catalytic or biological opportunities, while closed-shell d^10^ ions such as Zn(II) and Cd(II) tend to support ligand-centered emission because they lack low-energy d–d transitions that often quench fluorescence [61]. Nevertheless, even in d^10^ systems, a metal center should not be considered just as an inert framework, since coordination geometry, coordination number and metal-influenced packing can influence π–π interactions, rigidity, and relaxation mechanisms [62,63]. A recent matched-ligand pair based on 8-carboxymethoxyquinoline-2-carboxylate further illustrates this metal-dependent structural variation: the Ni(II) complex adopts κ^3^-N,O,O coordination in a six-coordinate octahedral environment, whereas the Zn(II) analogue uses κ^4^-N,O,O,O coordination and a seven-coordinate distorted pentagonal-bipyramidal geometry [64,65]. Because only structural and supramolecular evidence was established, this pair is best treated as a Level 1 comparative structural example rather than evidence of a descriptor–function relationship.

For redox-active metals like Cu, Co, Ni, Mn, Fe, etc., descriptor interpretation should be done with extra caution. A redox-active metal or the presence of a coordinatively accessible metal center does not by itself establish a catalytic or biological role. Supporting such assignments requires function-specific controls: free-ligand and metal-salt controls where appropriate, stability and speciation testing, leaching and post-reaction characterization for heterogeneous catalysis, and spectroscopic, electrochemical, or biological mechanistic evidence as applicable.

### 3.3. Coordination Systems Containing Lanthanides

Lanthanide carboxylates have distinctive coordination structures because of their relatively large ionic radii and high, variable coordination numbers [66], while their photophysical behavior can involve characteristic 4f–4f transitions and ligand-to-metal sensitization. Thus, ligand architecture and supramolecular packing become important parameters not only for structural but also for functional interpretation of the systems [67]. Emission from Eu(III) and Tb(III) is often sensitized through the ligand antenna effect, while Gd(III) analogues are commonly used to estimate ligand triplet-state energies. Analysis of metal–carboxylate descriptor–function relationships requires consideration of such features as lifetime, quantum yield, sensitization efficiency, coordinated solvent, ligand–metal distance, and ligand excited-state energy rather than merely listing the characteristic emission bands [68].

A reported Gd(III) complex containing a 4-acetylphenoxyacetate ligand and 1,10-phenanthroline exemplifies how lanthanide coordination descriptors, DFT-derived electronic outputs, and catalytic activity can be analyzed together without considering any single calculated parameter as proof of the mechanism [69].

### 3.4. Metal Nuclearity and Secondary Building Units

Metal nuclearity provides one link between coordination geometry and dimensional evolution. Metal–carboxylate systems can form mononuclear, dinuclear and polynuclear complexes including paddlewheels, metal–oxo–carboxylate clusters, and rod-like secondary building units [70]. Such motifs can be used as secondary building units for constructing frameworks [5].

Such a node-centered approach is valuable since it addresses questions concerning what metal–carboxylate motif is formed, how it is linked to ligand and whether it can be reproduced or varied. A node-centered approach is well correlated with analytic approaches of one-dimensional coordination polymers, MOF chemistry and metal carboxylate cluster architectures [71,72,73,74]. Nevertheless, the use of the SBU term is not appropriate for every recurrent motif in a low-dimensional coordination system; some recurrent motifs are structural characteristics that cannot be considered as elements of reticular construction.

### 3.5. Dimensional Evolution and Descriptor-Based Interpretation

Carboxylate-based coordination compounds should be discussed as a continuum from 0D complexes to 1D chains, 2D layers, and 3D frameworks. Across this continuum, changes in dimensionality may be accompanied by changes in connectivity, rigidity, packing, guest-incorporation ability, and possible energy- or charge-transfer pathways. However, this evolution does not reflect any hierarchy of functions. Discrete complexes provide a well-defined metal center for DFT calculations, ESP, docking, and molecular bioactivity studies, but functions may rely on packing, solubility and stability in the medium. Chains and layers include extended connectivity along with good structural interpretability and functions may additionally depend on interchain and interlayer interactions. Three-dimensional frameworks present the possibility of porosity and heterogeneity of functions. However, dimensionality alone does not establish functional performance; claims involving porosity, adsorption, or repeated use require activation stability, accessible-porosity measurements, adsorption data, cycling tests, and post-function structural characterization, as appropriate.

**Figure 3:**
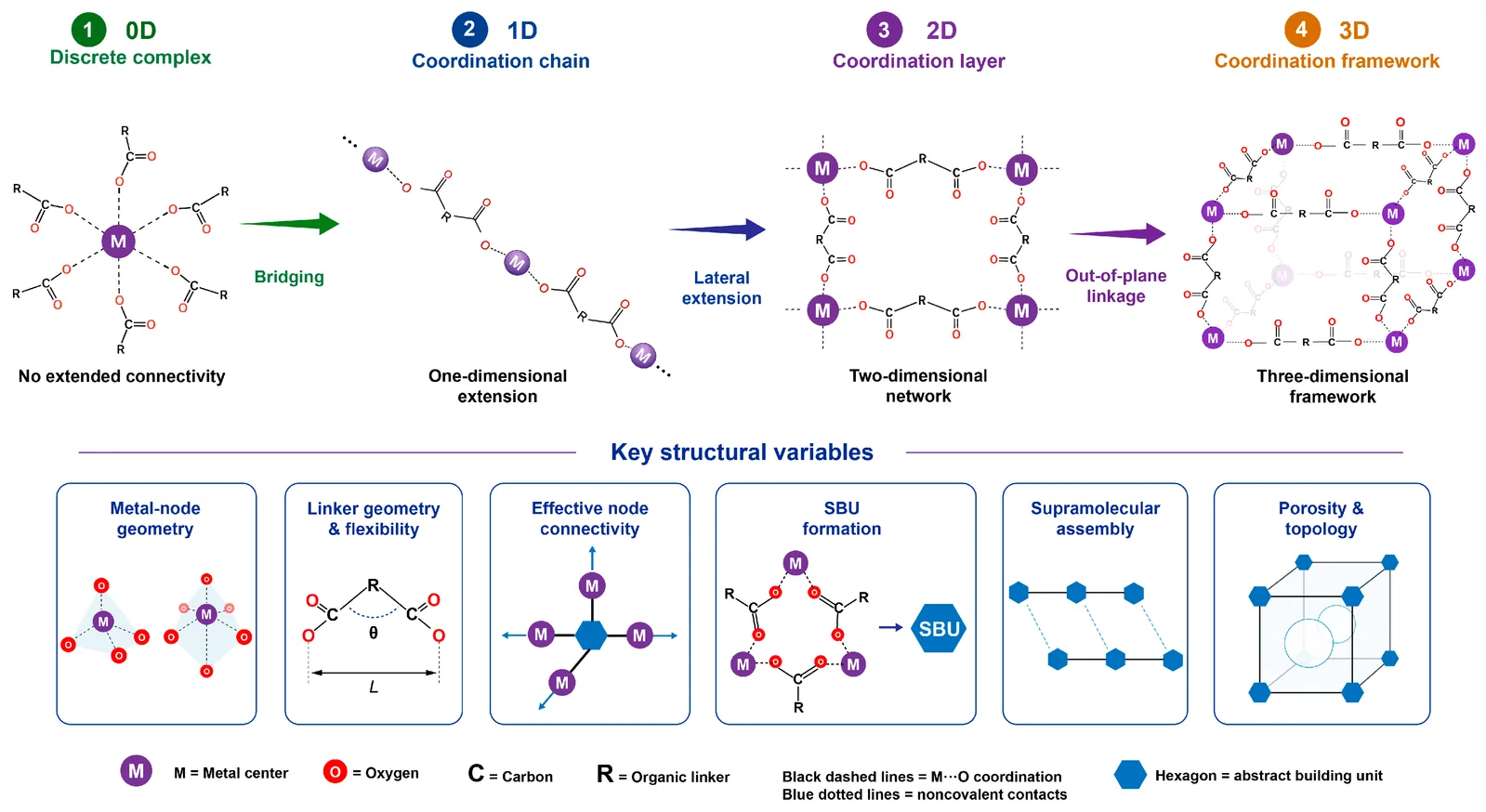
Structural dimensionality and associated factors in carboxylate-based coordination systems. Changes in metal–ligand connectivity can generate discrete complexes, one-dimensional coordination chains, two-dimensional coordination layers, and three-dimensional coordination frameworks. Metal-node geometry, linker geometry and flexibility, effective node connectivity, secondary building unit formation, and supramolecular assembly influence structural organization, whereas porosity and topology describe features of the resulting framework. Noncovalent contacts can organize preformed coordination entities into higher-order supramolecular assemblies but do not by themselves increase coordination dimensionality. The 0D–3D sequence represents a conceptual dimensionality continuum rather than a mandatory stepwise synthetic pathway. All structural motifs are schematic rather than specific crystallographically resolved structures; formal charges and carboxylate resonance are simplified for clarity.

Therefore, metal-center selection has to be discussed as a set of tendencies to generate particular descriptors rather than deterministic rules. Zn/Cd systems often support ligand-centered emission, lanthanides enable characteristic f–f luminescence and antenna-effect analysis, redox-active transition metals can support catalytic or bioactive behavior, and alkaline-earth metals may provide oxygen-rich Lewis acidic environments. A more general interpretation will be based on descriptors: metal-center selection can influence coordination geometry, nuclearity, electronic structure, rigidity and guest accessibility, and these factors can influence function under particular conditions. This formulation excludes the reduction of the review to the list of metal complexes and allows the transition to the Descriptor-to-Function Evidence Ladder in Section 4. The dimensional evolution from discrete complexes to three-dimensional frameworks is schematically represented in Figure 3.

## 4. From Crystal Structures to Descriptor-Based Evidence: Methods, Model Outputs, and Evidentiary Boundaries

Single-crystal X-ray diffraction has long been a foundation of coordination chemistry because it provides direct structural information about metal–ligand connectivity, coordination geometry, molecular conformation, and supramolecular packing. For carboxylate-ligand-based coordination polymers and metal complexes, this structural basis is especially relevant since carboxylate units can adopt several binding modes, auxiliary N/O-donor ligands can influence the coordination sphere, and even minor changes in the nature of the metal center, solvent, pH, counterion, or synthesis temperature can shift the structural outcome from a discrete compound to a chain, layer, or framework. Crystal structures should, thus, not be considered merely as final results to be published but as the primary sources from which chemically meaningful descriptors are extracted [28,75].

A descriptor becomes meaningful only when its physical meaning and evidentiary boundary are defined. Throughout this review, a method or model is distinguished from the quantity or feature obtained from it. Hirshfeld surface analysis is an intermolecular-contact partitioning and visualization methodology; its outputs include normalized contact distances, contact fractions, shape index, and curvedness. Density functional theory (DFT) is an electronic-structure method; depending on the model, its outputs can include molecular orbital energies, electrostatic-potential distributions, charge-density measures, band structures, densities of states, adsorption energies, or defect formation energies. Energy-framework analysis, grand canonical Monte Carlo simulation, and molecular docking are likewise methods or models, whereas pairwise interaction energies, occupancy/selectivity estimates, and docking poses or scores are outputs. These methods and outputs can support a hypothesis, but their presence does not by itself establish why a material is luminescent, catalytic, selective in adsorption, or bioactive.

Section 4, therefore, evaluates two linked questions. First, which experimentally derived or calculated outputs constitute meaningful structural, supramolecular, electronic, adsorption-related, or biological descriptors? Second, what can each descriptor support without a controlled comparison or function-specific validation? This separation prevents a computational or analytical method from being treated as a descriptor and prevents a descriptor from being treated as causal evidence merely because it has been calculated or visualized.

### 4.1. Primary Descriptors Related to Crystal-Structure Parameters

Many direct descriptors in carboxylate-containing coordination compounds are extracted from crystallographic models. They include metal–ligand bond lengths and angles, coordination number and geometry, metal–metal distances, ligand dihedral angles, coordination mode, hydrogen-bond geometry, aromatic-stacking distances, pore dimensions, void volume, and solvent-accessible volume. Crystallographic software such as OLEX2 supports the initial determination of an atomic model from diffraction intensities, subsequent refinement and validation, visualization, and extraction of these parameters [76]. These parameters describe the local and extended structural environment in which a function may emerge [75].

Reliable descriptor extraction depends on a high-quality crystallographic model. Data collection, initial crystal-structure determination, refinement, and validation are not merely technical steps; they govern how reliably subsequent structure–function correlations can be evaluated [77]. Inadequately modeled disorder or solvents, inappropriate hydrogen-atom treatment, and insufficient phase-purity evidence can compromise any descriptor–function inference [78].

At the level of local structure, coordination number and geometry reflect how a metal is embedded into its ligand environment. Tetrahedral Zn(II), octahedral Co(II), square-planar Cu(II), and high-coordinate lanthanide sites correspond to different electronic, steric, and supramolecular environments. For the carboxylate-based coordination compounds, the same metal ion may have different coordination geometries depending on monodentate, chelating, bridging and chelating-bridging coordination modes of the carboxylate fragment. This feature explains the possibility of structural regulation by carboxylate ligands but implies that coordination geometry should be interpreted as a comparative structural descriptor in function analysis rather than a standalone predictor.

At the level of the extended structure, the parameters such as discrete complexes and one-, two-, and three-dimensional structures have differences in connectivity, packing density, rigidity, guest accessibility, and continuity of coordination bonds. However, dimensionality per se is not the most informative parameter and should not be overinterpreted. A three-dimensional structure is not necessarily more functional than a discrete complex; in some cases, one-dimensional chains or low-dimensional assemblies may show stronger emission or biological response because of suitable ligand conformation, packing rigidity, solubility, or metal-centered electronic environment. Hence, dimensionality should be treated as a structural descriptor rather than as a direct predictor of functionality.

Non-covalent interactions constitute the next group of primary descriptors [79]. The oxygen atoms of the coordinating carboxylates, water molecules coordinated or existing in the lattice, or N-donor auxiliary ligands and aromatic rings can form O-H···O, C-H···O, N-H···O, C-H···π, and π–π interactions. Such interactions can stabilize packing and may contribute to ligand-conformation rigidity, solid-state luminescence, or guest recognition. Nevertheless, simple observation of such interactions in crystal structure cannot prove their participation in functionality. Functional interpretation of non-covalent interaction implies comparison with similar structures, photophysical or adsorption studies, guest response experiments, or evidence of changes of the interaction during functionality tests.

### 4.2. Descriptor Outputs from Hirshfeld Surface Analysis

Hirshfeld surface analysis is widely used to visualize and quantify intermolecular contacts [80,81,82,83]. It is the methodology; *d*_norm_ values and mapped surfaces, shape index, curvedness, and two-dimensional fingerprint contact fractions are the descriptor outputs. Recent spiro compounds provide an illustrative molecular-crystal application [84]. The outputs visualize close contacts and packing complementarity and quantify contact classes such as H···H, O···H/H···O, C···H/H···C, N···H/H···N, C···C, and metal-involved contacts.

For coordination complexes and coordination polymers containing carboxylate anions, Hirshfeld surface analysis is valuable due to the significant role of weak interactions in the determination of the supramolecular architecture [85]. Carboxylate oxygen atoms frequently take part in hydrogen bonding, aromatic ligand backbones promote C–H···π and π–π interactions, and additional N-donor ligands are capable of generating extended packing motifs. The use of Hirshfeld surfaces for describing crystal packing is more systematic than selecting some specific hydrogen bonds and stacking distances.

However, the evidentiary relevance of Hirshfeld-surface outputs depends on the claim being made. A high percentage of O···H/H···O contacts indicates frequent occurrence of such contacts, but it does not prove that these contacts dominate the packing energy. High H···H contribution can result from the large surface area of the ligand rather than functional importance. Red areas on a *d*_norm_ surface indicate contacts shorter than the corresponding sums of van der Waals radii but not necessarily controlling sensing, catalysis, adsorption, or biological response.

This fact should be taken into account during functional studies. Suggested host–guest interactions based on Hirshfeld surface analysis can be quite reasonable, but they require function-specific evidence, such as quenching constants, spectral overlap analysis, fluorescence lifetimes, quantum yields, competitive experiments, or structural evidence of the guest binding. In case of biological applications, Hirshfeld contacts are able to provide the description of packing and intermolecular stabilization but not the proof of DNA binding, protein interaction, cellular uptake, or cytotoxic mechanism. The contact and surface outputs of Hirshfeld analysis should therefore be treated as supramolecular descriptors that can support mechanistic interpretation, not as substitutes for mechanistic evidence.

### 4.3. Interaction-Energy Descriptors from Energy-Framework Analysis

For molecular crystals and suitably partitionable coordination compounds, energy-framework analysis is a model-based crystal-packing methodology that calculates approximate pairwise interaction energies between molecular or ionic units and represents the magnitudes and directions of those interactions as a three-dimensional network [86]. The calculated total and component energies—commonly electrostatic, polarization, dispersion, and exchange-repulsion terms—and the resulting energy-network topology are the descriptor outputs. This approach extends contact geometry by asking which pairwise interactions are energetically important, not merely frequent.

Energy framework analysis is informative for molecular carboxylate complexes and other suitably partitionable coordination compounds whose crystal packing involves both strong and directional hydrogen bonds and aromatic contacts. A packing motif that looks visually dominated by π–π stacking can actually be stabilized by electrostatic O–H···O or C–H···O interactions, while a crystal packing having high H···H contact contribution in fingerprint plots can be energetically insignificant. Therefore, energy frameworks can help to avoid oversimplifying the results and to obtain a more physically meaningful picture of supramolecular stabilization.

Nevertheless, the interaction energies and network topology obtained from energy-framework analysis remain model-dependent packing descriptors. They can rationalize the stability and directionality of a packing motif, but they cannot by themselves establish catalytic activity, sensing selectivity, adsorption capacity, or biological mechanism. If a packing network is claimed to enhance solid-state luminescence by increasing rigidity, the claim requires relevant measurements, such as photoluminescence quantum yields, lifetimes, temperature-dependent emission, or comparison with a less rigid analogue.

### 4.4. Electronic Descriptors from Molecular, Cluster, and Periodic DFT Models

DFT is an electronic-structure methodology rather than a descriptor [87,88]. For discrete complexes and finite clusters, outputs can include optimized geometry, molecular orbital energies/localization, HOMO–LUMO gaps, molecular electrostatic potential, and population-derived charges. Such local outputs are relevant, for example, to photochromic coordination polymers [89] or metal-complex-mediated O_2_ activation [90]. For periodic coordination polymers and MOFs, band structure, DOS/PDOS, periodic charge-density redistribution, adsorption energies, and defect formation energies are more representative when extended connectivity is central [91]. Reliable use requires reporting the functional, basis set or plane-wave cutoff, dispersion, solvation or boundary treatment, and the relationship between crystallographic and optimized structures [92,93]; broader MOF-modeling considerations are reviewed in [94].

For weakly bound or flexible systems, model choice must follow the claim. Molecular models suit established discrete solution species; finite clusters can probe local sites if termination, charge, and boundaries are justified; periodic models are preferable for extended states, transport, lattice-wide charge redistribution, adsorption, or defects. Dispersion, solvent, flexibility, loading, spin, and oxidation state must also be considered [19,94,95,96].

An electrostatic potential map can be used to locate the electron-rich and electron-poor regions of the molecule [97]. In carboxylate-containing systems, negative potential is usually observed in the vicinity of carboxylate oxygen, heteroatoms, or coordinated solvent molecules, whereas positive potential is observed at hydrogen atoms, metal-adjacent areas or electron-poor aromatic fragments. These data can be used to rationalize potential hydrogen-bonding, guest-binding, or biomolecule-interacting sites. However, the presence of an ESP hotspot does not mean that it is a binding site. Binding involves not only geometrical availability of the site but also solvent compatibility, thermodynamic preference and competition with other substances. Thus, ESP-derived interpretation should be supported by corresponding experimental evidence from guest-binding or adsorption studies, titration, spectroscopy and crystallographic evidence of host–guest interactions.

The HOMO–LUMO gap is another frequently-used descriptor. Its small value is interpreted as high chemical reactivity, easy charge transfer or strong electronic response. This interpretation can be helpful as a trend in a comparable series, but it is widely over-extended. Solid-state luminescence is governed by excited state dynamics, ligand rigidity, aggregation, crystal packing, non-radiative decay channels and metal-centered or ligand-centered transition. Catalytic activity depends on substrate adsorption, transition state stabilization, mass transport and availability of the active site. Biological activity implies cellular uptake, hydrolytic stability, redox cycling, ROS production and target engagement. HOMO–LUMO gap alone cannot prove any of these phenomena.

Nevertheless, the distribution of frontier orbitals can give some clues. If the HOMO is located mainly on a carboxylate ligand and LUMO on an auxiliary N-donor ligand, a low-energy excitation may have intraligand or ligand-to-ligand charge-transfer character. If the frontier orbitals are localized on both metal and ligand centers, metal-to-ligand or ligand-to-metal charge-transfer contributions may be possible [98]. Localization of orbitals on aromatic ligands may be consistent with ligand-centered excited-state character or π-conjugation effects. These assignments are even more convincing if supported by UV–vis absorption, photoluminescence spectra, lifetime measurements, electrochemical data and, if possible, time-dependent DFT calculations.

Approximate atomic charges obtained from Mulliken population analysis, natural population analysis within the NBO framework, Hirshfeld charge partitioning, and related schemes can provide additional information about charge distribution [99]. These data are useful for comparing the properties of the similar molecules calculated with the same method, but their absolute values depend on the computational method and cannot be considered as experimental. Positive or negative calculated charge on an atom can suggest potential interaction sites but this does not define the active site in catalysis or the biological target in cells.

### 4.5. Function-Specific and Data-Assisted Descriptors

Apart from general structure/electronic descriptors, function-specific descriptors have been used to analyze luminescence, sensing, catalysis, adsorption/separation, small-molecule reactivity, and bioactivity [100,101]. In luminescent complexes, time-dependent DFT can help assign specific absorption bands and possible excitation paths. In combination with experimental photoluminescence spectra, lifetime and quantum yield measurements can help distinguish ligand-centered transitions, charge transfer states, and metal-based emissions. For lanthanide complexes, descriptors related to ligand triplet state energy, antenna efficiency, ligand-to-metal energy transfer, and water-quenching in the metal environment are particularly important. The lack of this type of information prevents a well-supported discussion of sensitization or energy transfer.

In the context of adsorption and separation, grand canonical Monte Carlo simulations and other molecular modeling approaches can predict adsorption sites, guest molecule distribution, adsorption energy, and selectivity trends [102]. These methods are suitable for porous coordination polymers and MOF-like materials, where an experimental crystal structure provides a starting pore model whose relevance after activation must be checked. Nevertheless, predicted performance in adsorption depends on force-field parameters, assumptions regarding framework rigidity [103], charge assignment, pore activation, defect consideration, and presence/absence of solvent molecules. Therefore, GCMC predictions should be benchmarked against experimental isotherms; separation claims additionally require competitive adsorption and, ideally, breakthrough testing, with cycling and humidity/stability assessment as relevant.

In bioactivity research, molecular docking is often employed to suggest putative interactions of metal complexes with biomolecular targets such as DNA, proteins or enzymes. Molecular docking can suggest binding modes, hydrogen bonding, hydrophobic interactions, and relative docking-score trends. This method is helpful for generating mechanistic hypotheses. However, molecular docking scores cannot be used to claim antitumor or antibacterial mechanisms of action. In biological environments, the metal complexes can undergo ligand substitution, hydrolysis, redox conversion, protein binding, aggregation, or dissociation of metals. Hence, docking-based interpretations need biological validation, including cytotoxicity against normal cells, cellular uptake studies, ROS generation assays, apoptosis or cell-cycle assays, DNA/enzyme binding experiments, and stability tests in biological media.

A new trend relates to data-driven structural descriptors. Structural databases and machine-learning methods can encode metal identity, ligand geometry and topology, pore dimensions, charge distribution, and electronic quantities to screen coordination systems or MOFs for target properties [104]. Here again, the model is the method, whereas learned features, predictions, uncertainty estimates, and applicability-domain measures are outputs. This trend can support prospective design rather than only post hoc interpretation [105,106]. However, a model is informative only when trained and evaluated on curated, comparable, chemically meaningful data; predictive correlation does not automatically establish chemical causality, and prospective experimental validation remains the strongest evidence.

However, some references can be made to crystalline systems outside strictly carboxylate-based coordination polymers. Hydrogen-bonded organic frameworks and salt cocrystals illustrate how Hirshfeld-surface outputs and DFT-derived electronic descriptors can be related to hydrogen-bond networks and electrostatic recognition [107,108]. Spirocyclic compounds illustrate combined use of Hirshfeld-surface outputs, MEP/NBO outputs, and thermodynamic, photophysical, and NLO descriptors [109,110,111,112]. A donor–acceptor coronene/Ag(I) pyrazolate cocrystal further demonstrates integration of packing, spectroscopy, and DFT analysis [113]. These systems are methodological analogies rather than direct evidence for carboxylate coordination chemistry. By contrast, the water-stable Zn-MOF [114] is an in-scope example combining crystallographic characterization with multianalyte luminescence sensing in water.

The same approach should be followed in the case of porous and catalytic coordination systems. Cd(II) complex with catalytic testing or Cu(II) MOF with DFT/GCMC analysis can support adsorption or catalytic reasoning only when simulation outputs, accessible structural features, and experimental performance are evaluated together [115,116]. Hence, the power of function-specific descriptor depends on its connection with the particular functional question and corresponding experiment.

### 4.6. Common Over-Interpretations and Minimum Validation Requirements

The greater availability of descriptor-generating methods has improved structural interpretation, but it has also encouraged a recurrent evidence gap: synthesis, single-crystal structure determination, a Hirshfeld or DFT analysis, and one functional test may be reported in the same study, even though the calculated or mapped output is not experimentally linked to the function. The calculations or contact analysis may be technically valid while the descriptor–function explanation remains post hoc. Therefore, the issue evaluated here is not whether a method was performed correctly but whether the resulting descriptor supports the strength of the functional claim.

The first common type of over-interpretation is interpreting contact abundance as functional importance. Fingerprint plots may show that H···H contacts occupy the highest fraction of the Hirshfeld surface area, but this does not mean that H···H contacts dictate luminescence, catalytic activity, adsorption, or cytotoxicity. The second common over-interpretation is using the presence of red spots on a *d*_norm_ plot as evidence for the mechanism of guest recognition or biological interaction. Red spots indicate contacts shorter than the relevant van der Waals-distance reference in the analyzed crystal; they do not prove that the same contacts form in sensing, catalysis, adsorption, or biological experiments.

A third common over-interpretation involves electronic descriptors. HOMO–LUMO gaps are used to explain trends of activity, but lower gaps do not necessarily imply superior function. For luminescence, factors such as nonradiative relaxation processes, rigidity of molecules, molecular aggregation, and crystal packing may become decisive. For catalysis, accessibility of the active site, substrate adsorption, stabilization of transition states, and stability in reaction conditions are necessary. Bioactivity is determined by uptake, speciation, redox properties, and biochemical mechanism. ESP plots and charge distributions can identify possible interaction regions but are unable to provide evidence of binding or biological selectivity.

A fourth common over-interpretation is treating simulation and docking data as experimental evidence. GCMC simulations may predict possible adsorption sites, but they are no substitute for isotherms, competition, regeneration, and breakthrough experiments. Molecular docking may suggest plausible biomolecular interactions, but it provides no evidence of cellular uptake, medium stability, activation of the pathway, or selectivity of action. Computational predictions help with candidate selection but need experimental validation.

A fifth common over-interpretation is the absence of controlled comparison. If only one compound is investigated, it is generally difficult to isolate which factor—carboxylate ligand, metal ion, auxiliary ligand, structural dimensionality, packing contacts, defects, particle size, or testing conditions—is responsible for the functional behavior. More substantial claims can be made if one makes a comparative series, e.g., the same ligand with different metal ions, the same metal ion with different ligands, the same metal–ligand pair with different auxiliary ligands, or structurally similar molecules with different dimensionalities. Absence of such comparison leaves descriptor–function relationships at the level of hypotheses.

Concrete cases illustrate these boundaries without judging overall study quality. The Zn-MOF study [114] establishes crystallographic identity and multianalyte sensing, but its proposed quenching explanation is not discriminated by lifetime measurements or matched analogues. The Cd(II) study [115] reports Hirshfeld-surface packing outputs and catalytic activity, but these alone do not identify the active species. The Cu(II) MOF study [116] uses DFT/GCMC to support an adsorption hypothesis, but simulated uptake or sites are not experimental adsorption performance. Docking comparisons [109,112] likewise require target-engagement evidence. Level 2 here applies only to these descriptor–function inferences, not to the papers or their reported performance.

Therefore, descriptor-generating methods must be used with explicit evidentiary boundaries. A descriptor can formulate a hypothesis, support a controlled trend, or contribute to a mechanistic interpretation, but it becomes compelling only when paired with validation appropriate to the claim. Luminescent sensing may require selectivity, limit of detection, Stern–Volmer analysis, lifetime and quantum-yield measurements, assessment of spectral overlap and inner-filter effects, and guest-induced changes; catalysis may require kinetics, controls, substrate scope, turnover metrics, recycling, leaching tests, and post-reaction characterization; adsorption may require activation verification, isotherms, competitive or mixture data, regeneration, humidity testing, and stability; and bioactivity may require normal-cell controls, dose–response analysis, medium stability/speciation, uptake, and mechanism-supporting assays. Table 2 separates methods from descriptor outputs and summarizes their physical meaning, evidentiary limits, literature illustrations, and claim-appropriate validation.

### 4.7. Descriptor-to-Function Evidence Ladder

The Descriptor-to-Function Evidence Ladder is proposed as a claim-centered operational framework. Its unit of assessment is one explicit relation between a defined descriptor and a defined functional response—not the overall quality, novelty, or importance of a paper. The same paper can therefore receive different levels for different claims. For example, an excellent crystallographic study can appropriately remain at Level 0 or Level 1 if it did not seek to test a function, whereas a multifunctional material may require separate ratings for sensing, adsorption, and bioactivity. Its proposed added value is a common set of decision rules, conservative caps for missing prerequisites, and an explicit rationale across functional domains. These rules reduce, but do not eliminate, interpretive judgment; inter-rater performance has not yet been established.

Level 0 is structure-only reporting: a validated structure or material identity is established, but no descriptor–function relation is tested.

Level 1 is descriptive use: a method-derived descriptor is reported, but no linked functional hypothesis is tested; this is not a criticism of study quality.

Level 2 is co-reporting or post hoc association: a descriptor and response are reported for the same system without matched descriptor variation or a discriminating intervention.

Level 3 requires a matched series or targeted intervention in which descriptor variation tracks a reproducible response under matched conditions with major design variables controlled.

Level 4 adds claim-specific controls and orthogonal evidence that discriminate leading alternatives; it supports a mechanism-linked interpretation but not definitive proof unless alternatives are excluded.

Level 5 requires a descriptor-based prediction specified before a new synthesis or test and then confirmed by an independent experiment; retrospective fitting does not qualify. Predictive confirmation does not by itself establish mechanism, which must satisfy Level 4 criteria for a separately stated mechanistic claim.

**Figure 4:**
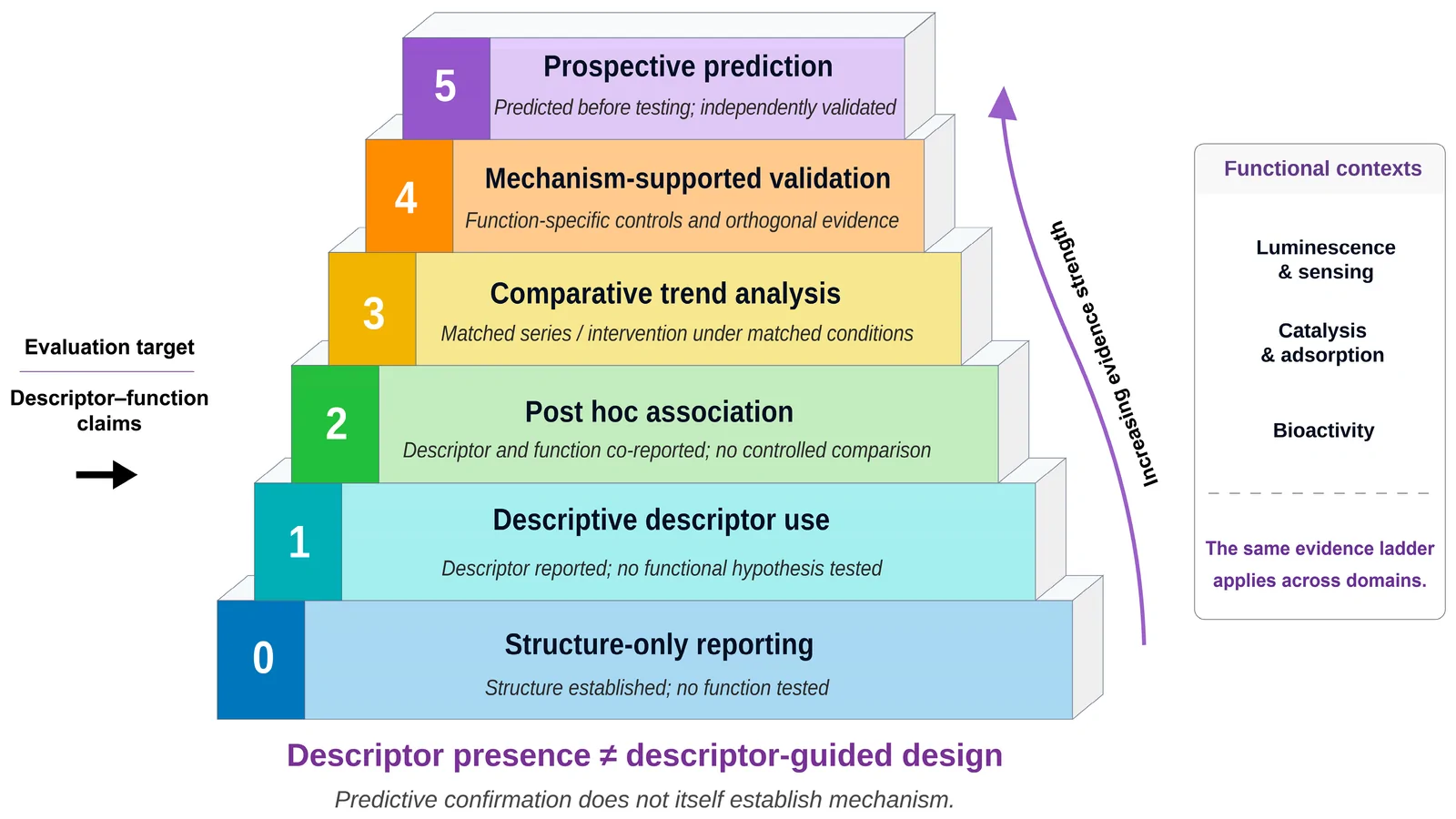
Descriptor-to-Function Evidence Ladder for evaluating individual descriptor–function claims. The six-level framework progresses from structure-only reporting and descriptive descriptor use to post hoc association, controlled comparative trends, mechanism-supporting validation, and prospective prediction followed by experimental confirmation. The ladder does not rank the overall quality or importance of a study. Predictive and mechanistic claims are assessed separately. Operational assignment criteria, conservative boundary rules, and examples of what does not suffice are provided in Table 3.

Figure 4 summarizes the six levels, and Table 3 provides the operational assignment rules. Classification proceeds claim by claim: the highest level is assigned only when all necessary conditions are supported by the report. If a prerequisite is missing or a boundary is ambiguous, the lower level is retained and the limiting reason is stated. This conservative rule distinguishes structural reporting, descriptive interpretation, co-reporting, controlled comparison, mechanism-supporting validation, and prospective confirmation without devaluing studies whose objectives appropriately lie at Levels 0 or 1.

## 5. Function-Oriented Design

This section applies the Descriptor-to-Function Evidence Ladder across three functional domains: luminescence and sensing; catalysis, adsorption, and small-molecule transformations; and bioactivity and biomedical relevance.

### 5.1. Luminescence and Sensing

The two topics are especially suited for this approach as optical responses are influenced by many factors including the rigidity of ligands, the metal itself, coordination, crystal packing [117], host–guest chemistry, framework stability and electronic transition pathways. Meanwhile, the area is susceptible to an overinterpretation of descriptors, as the enhancement or quenching of emission is often explained with hydrogen bonding, π–π interactions, charge transfer, pore confinement and analyte interactions without sufficient mechanistic distinction [118]. Thus, luminescent systems are used in this review to assess how structural variables, supramolecular descriptors and photophysical measurements are used to support descriptor-guided function-oriented design.

A valid claim about luminescence or sensing ability should correlate the measured optical properties with variables such as ligand conjugation, the rigidity of the complex, the center of the emission (metal versus ligand), presence of accessible guest sites, and interactions with the analyte [119,120,121]. The observation of enhanced emission, color changes, or analyte selectivity offers limited evidence. For better understanding, in some cases, the measurement should be compared with the free ligand in solution or related structures, together with absorption and emission spectra, quantum yield, lifetime, Stern–Volmer plots, spectral overlap, competitive sensing, reversibility, testing in real samples or matrix effects, and PXRD analysis or other means of showing structural integrity [61,122].

#### 5.1.1. Optical Origins and d^10^ Metal Systems

The emission in coordination systems containing carboxylate ligands may be attributed to ligand-centered transitions, ligand-to-metal or metal-to-ligand charge-transfers, guest-induced effects, metal-centered emission, or sensitization of lanthanide emission. For many closed-shell d^10^ Zn(II) and Cd(II) coordination systems [123], coordination may facilitate ligand-centered emission through the rigidification of the ligand, inhibition of non-radiative decay, modification of π-conjugation, and packing of crystals [124,125]. Carboxylate ligands can easily exhibit such behavior due to their combination of hard O-donors in coordination, aromatic cores, flexible or semi-rigid spacers, and, often, N-donor auxiliary ligands [126,127].

While such a rationale sounds chemically plausible, it must not be accepted automatically. The presence of a d^10^ metal ion is not sufficient evidence for ligand-centered emission, just as the presence of π–π stacking is not proof of aggregation-enhanced emission. Phenoxyacetic acid derivatives coordinated with Cd(II) demonstrate that fact: coordination of the carboxylates, presence of N-donor auxiliary ligands, supramolecular contacts, Hirshfeld analysis, and emission properties may be discussed together, but the functional significance of the descriptors will remain mostly descriptive without comparison to free ligands, related complexes, lifetimes, quantum yields, and TD-DFT assignments [128,129].

This is why the evidence for the origin of the emission in d^10^ metal systems critically relies on the separation of molecular from crystalline effects. Emission in solid-state may differ from emission in solution due to aggregation, conformation of the ligand, interchain contacts, solvent inclusion, and state of the particles, which means that comparative analysis of the free ligand, synthesized crystal, ground crystals, crystals exposed to the solvent, and structurally related systems is useful. Such a comparative analysis is not obligatory in each investigation, but it helps avoid automatically attributing all intensity changes to coordination-induced rigidity and π-stacking.

#### 5.1.2. Lanthanide Systems and Antenna-Effect Evidence

A unique luminescence property of lanthanide carboxylates relates to the possibility of f–f emission sensitization through ligand absorption and ligand-to-metal energy transfer [130]. Eu(III) and Tb(III) derivatives are especially common since their red and green emissions have a diagnostic character [131]. Antenna role may be played by carboxylate ligands, and auxiliary ligands and supramolecular packing may affect ligand triplet-state energy, metal coordination sphere, nonradiative quenching and guest accessibility [68].

Observing characteristic Eu(III) or Tb(III) emissions alone is not enough to prove the antenna mechanism. Stronger evidence may include, as applicable, the analysis of excitation spectra, emission lifetimes, quantum yields, study of coordinated solvent or water quenching, and estimation of ligand triplet-state energy with respect to the emitting lanthanide level. In sensing applications, analyte-induced enhancement or quenching should be related to the possible mechanism, such as energy-transfer modulation, analyte coordination, framework destabilization, competitive absorption, or nonradiative decay change [132].

For lanthanide–carboxylate frameworks, such discrimination is particularly important when sensing is inferred from emission quenching or altered sensitization [133]. Decreases in Eu(III) or Tb(III) emission may be explained by analyte absorption, interruption of ligand-to-metal energy transfer [134], competitive coordination, water-induced quenching, framework instability, or dilution or inner-filter effects. Turn-on emission can be caused by rigidity increase, removal of quenching solvent, or analyte-induced energy transfer [135]. Thus, the descriptor claim should indicate exactly what stage of the antenna process is influenced, not assign all response behavior to the host–guest interaction.

#### 5.1.3. Luminescent Sensing: Response Versus Mechanism

Sensing studies using luminescent frameworks should be organized in accordance with the response mechanism and its evidentiary character [136,137,138]. Sensing of metal ions, anions, nitroaromatics, solvents, gases, and biomarkers always involves signal response, but the mechanism of that response may significantly vary [139,140,141]. Signal change can arise from photoinduced electron transfer, energy transfer, static or dynamic quenching, apparent intensity loss through inner-filter effects, competitive absorption, guest binding, coordination-site blocking, framework deformation, or partial decomposition [118,142]. Recent examples include modular multivariate MOFs and two-dimensional luminescent coordination polymers for aqueous-pollutant sensing [143,144]. Therefore, evaluation should address selectivity, interference tolerance, reproducibility, and reversibility, together with binding or interaction evidence, lifetime or absorbance data, and framework-stability tests as appropriate to the proposed mechanism [10].

These systems are included for distinct evidentiary reasons. The related Zn(II)/Cd(II) d^10^ MOFs [123] illustrate confounding: metal substitution coincides with changes in coordination and topology, so they are not a matched Level 3 series and the metal/structure–sensing inference remains Level 2. The single Cd-MOF [145] and lanthanide MOF/membrane sensor [146] report sensing performance, but descriptor–mechanism inferences likewise remain Level 2 without controlled variation or discriminating evidence. These examples delimit rather than establish a transferable rule.

#### 5.1.4. Turn-On Probes and Biologically Relevant Sensing

Turn-on probes and biological-matrix sensing require stricter validation [147,148,149,150]. A cyclodextrin-based material coupling Ag^+^ detection with Congo-red adsorption [151] shows why each function requires separate validation. An Mn(II) coordination-polymer sensor for urinary biomarkers [127] further highlights matrix effects and biological-sample validation. The cyclodextrin-based H_2_S nanotube [152] remains a boundary example for cell imaging.

It also becomes clear how to draw a line between sensing and broader bioactivity. Detection in a biological matrix or a cellular response does not necessarily mean that the material is a therapeutic agent or biomaterial, so the material needs cell viability testing, imaging controls, medium stability, and selectivity of the response. In a similar way, the combination of gas adsorption, fluorescent response, and antibacterial response of the porous system should not be explained by a single universal structure–function explanation. The optical function, adsorption function, and biological function each require their own validation.

#### 5.1.5. Descriptor Interpretation and Evidence-Chain Evaluation

Hirshfeld surface analysis maps close intermolecular contacts and quantifies their contributions to the molecular surface. O···H/H···O, C···H/H···C, and C···C contacts may be consistent with hydrogen-bonding, C–H···π, and aromatic-stacking motifs, respectively, but contact percentages alone neither establish interaction energies nor report excited-state dynamics. Mechanistic claims of quenching or enhancement should be evaluated using claim-appropriate evidence such as absorption–emission relationships, lifetime changes, Stern–Volmer behavior, and matched solid-state/solution-state or analogue comparisons [13,153]. DFT-based ESP analysis can identify electrostatic regions, and frontier-orbital localization can suggest possible donor/acceptor character, while TD-DFT can help assign ligand-centered or charge-transfer transitions. However, calculated maps or orbital diagrams do not prove a sensing mechanism; their evidentiary value depends on interpretation alongside relevant spectra, guest-response experiments, framework-stability evidence, and controlled structural series [154].

Thus, luminescent behavior and sensing should be interpreted via evidence chain and not via a single descriptor. Level 1 studies report descriptors without testing a functional hypothesis, whereas Level 2 studies co-report emission or sensing behavior with structural or electronic descriptors but lack controlled comparison. Level 3 studies evaluate descriptor–response trends through controlled comparative series. Level 4 studies add function-specific controls and mechanism-supporting photophysical or structural evidence. Level 5 studies use descriptors prospectively to predict an emission or sensing response and then test that prediction experimentally. The goal here is not to reject the descriptive photoluminescence studies, which remain important for discovering novel luminescent structures, but to differentiate those from experiments establishing descriptor–function relationships. Luminescence and sensing clearly illustrate the central idea of this review on how the descriptors become meaningful via comparative series, photophysical rigor, analyte-specific controls, and mechanism validation. Representative luminescence and sensing studies are evaluated according to the Descriptor-to-Function Evidence Ladder in Table 4.

### 5.2. Catalysis, Adsorption, and Small-Molecule Transformations

Catalysis, adsorption, and small-molecule transformations provide a demanding test of descriptor–function relationships because the relevant structural descriptors must remain meaningful under working conditions [157]. In these systems, a crystallographically defined metal site, pore environment, ligand functionality, or supramolecular contact is not automatically an active site, adsorption site, or separation origin. It becomes functionally meaningful only when linked, as applicable, to substrate access, reaction or adsorption behavior, selectivity, stability, regeneration, and post-function structural retention. The Descriptor-to-Function Evidence Ladder is therefore useful for distinguishing structure-based hypotheses from validated catalytic or adsorption mechanisms.

This section is intentionally narrower than a general review of MOF catalysis [158,159,160,161], energy conversion [162,163], or porous-material separation. Its focus is on how carboxylate-based coordination systems use metal–carboxylate nodes, ligand environments, pore structures, and electronic descriptors to support catalytic [164,165,166,167] and adsorption claims [168]. Energy electrocatalysis is outside the main scope. For example, MOF-based electrochemical CO_2_ reduction requires a separate performance framework involving overpotential, Faradaic efficiency, current density, product selectivity, and stability [162,163,169,170]. Such examples are mentioned only as boundary cases when they help clarify descriptor–evidence standards.

#### 5.2.1. Catalytic Descriptors and Evidence Requirements

Contributions to catalysis by the metal–carboxylate coordination system could include, but would not be limited to, Lewis acidic metal sites, redox-active centers, coordinatively unsaturated metal nodes, ligand-assisted effects on electronic properties, immobilization of an active species, or a microenvironment defined by pores. Such structural features may be chemically significant, but alone they are not sufficient for catalytic function. A metal node proposed as the active site should be shown to be accessible to the substrate and sufficiently stable under reaction conditions, and a redox-active center should not be assigned a catalytic role without evidence of participation in the catalytic cycle. A framework feature becomes mechanistically meaningful only when its role can be distinguished from those of leached metal species, nanoparticles, residual precursors, or homogeneous transformation products [171].

Catalytic descriptors need to be applied in connection with mechanistic considerations. Hirshfeld surface analysis can characterize close intermolecular contacts and packing motifs, but it cannot identify a catalytic active site. Electrostatic-potential (ESP) maps may suggest polar regions for interaction with substrates, but they do not establish selective adsorption geometry. Frontier-orbital descriptors may indicate electronic trends but do not determine reaction kinetics. DFT calculations are most informative when their outputs address plausible substrate geometries, intermediates, transition states, and energy profiles rather than only ground-state optimization. The same applies to pore size, presence of open metal sites, and topology of the framework. Only if substrate access, turnover behavior, and structural stability are demonstrated do they become functionally meaningful catalytic descriptors [172].

Stronger catalytic claims require claim-appropriate comparisons and validation. A structural series, in which one feature is varied while others remain similar—different metal ions but same ligand, different ligands but same metal ion, framework before and after post-synthetic modification, related systems under varying pore environment conditions—can take us from post hoc rationalization toward structure–activity correlations [154,173]. Mechanism-based interpretation requires adequate blanks, appropriate free ligand and metal salt controls if needed, kinetic information, substrate scope, turnover metrics, heterogeneity tests, leaching study, recycling, and post-reaction powder X-ray diffraction (PXRD), IR spectroscopy, X-ray photoelectron spectroscopy (XPS), microscopy, and elemental analysis [160,174].

#### 5.2.2. Photocatalytic CO_2_ Reduction and Organic Transformations

Photocatalytic CO_2_ reduction is retained here only as a descriptor–evidence test case, not as a major energy-conversion topic [175,176]. Broader MOF/COF remediation and CO_2_-conversion literature provide contextual background [177,178]. This is useful because it involves light absorption, charge separation [179], CO_2_ binding, product selectivity, and catalyst stability [180]. A crystal structure, ESP map, Hirshfeld analysis, or frontier-orbital distribution may suggest possible CO_2_ interaction or charge-transfer pathways, but these descriptors cannot prove CO_2_ reduction activity by themselves. Stronger evidence requires blank experiments without catalyst, light, or CO_2_, product quantification, carbon-source verification where possible, selectivity analysis, recycling, and post-reaction structural checks [181].

Ca(II), Cu(II), and dinuclear Gd(III) carboxylate-based systems reported for photocatalytic CO_2_ reduction can therefore be discussed according to evidence completeness rather than apparent product formation alone [182,183,184]. The cited single-system studies primarily provide Level 2 evidence because they co-report crystal structures or calculated descriptors with product formation but do not establish a controlled descriptor–response trend. Such studies approach Level 4 only when claim-appropriate controls, calibrated product verification, carbon-source verification, catalyst-stability evidence, and mechanism-supporting experiments are included [185]. This treatment preserves CO_2_ photoreduction as a relevant descriptor example while preventing the chapter from becoming an energy-catalysis review.

Small-molecule catalytic reactions, such as benzylic alcohol oxidation [186,187,188] and related organic transformations [189,190,191,192], provide a more suitable platform for the present review because they can often be evaluated using conversion, selectivity, turnover metrics, substrate scope, recyclability, and heterogeneity tests. Alkaline-earth, transition-metal, and lanthanide carboxylate systems may be interpreted in terms of Lewis acidity, substrate access, framework extension, or ligand-assisted electronic effects, but the catalytic origin still requires control experiments and post-reaction structural verification. Recent Zn(II), Co(II), Ni(II), and Cd(II) coordination polymers assembled from a flexible tricarboxylate and auxiliary N-donor linkers are, therefore, useful as representative examples of how crystallographic definition and catalytic testing can be connected, provided that the claim remains consistent with the available evidence [9].

MOF-supported metal or nanoparticle catalysts require additional caution. In the cited Au–Pt/UiO-67 and Pt–Co/MIL-100(Fe) systems, the framework primarily acts as a support, dispersant, or confinement environment rather than as the intrinsic catalytic center [193,194]. Such cases should be described as composite or immobilized catalyst examples, unless the specific catalytic role of the coordination framework is independently demonstrated.

#### 5.2.3. Adsorption and Separation: Pore Descriptors Versus Practical Evidence

Adsorption and separation offer a more direct route from structure to function, although the same caution about evidence evaluation applies here as well [195]. Useful descriptors include pore size, pore shape [196], accessible surface area, pore volume [197], open metal sites, functional groups, adsorption enthalpy, Henry’s constant, IAST selectivity, and GCMC-predicted binding sites [198,199]. However, crystallographic void volume does not necessarily imply permanent porosity, high single-component uptake does not imply separation, high adsorption enthalpy can make regeneration difficult, and simulated binding sites need comparison with experimental adsorption [200,201].

CO_2_ uptake and membrane-based carbon capture are represented by composite, review, and mixed-matrix-membrane studies [202,203,204]. Light-hydrocarbon separation [205] and one-step ethylene purification are represented by [206,207,208,209]. Solvent-regulated C_2_H_2_/CO_2_ [210] and CO_2_/CH_4_ separation [208,211], dicarboxylate-CP adsorption, and carboxyl-functionalized pore-space-partitioned MOFs provide more specific structure–separation examples [212,213,214]. ML-guided propane selectivity and MOF membrane design are distinct data/process routes [215,216,217]. A combination of structure with N_2_ sorption and single-component uptake usually provides Level 2 evidence of adsorption potential. Comparative pore engineering or functional group modification can bring evidence up to Level 3 provided performance trends are established under identical conditions. Depending on the intended separation and operating conditions, Level 4 evidence may include competitive or mixture adsorption, breakthrough measurements, cycling and regeneration, humidity tests, and post-adsorption structural characterization. For membrane-based separation [218], adsorption data alone are insufficient; membrane continuity and interfacial compatibility, together with permeance or permeability, selectivity, and operating stability, should also be evaluated [219,220]. Recent progress in COF-based membranes provides a useful out-of-scope process comparator, because it likewise shows that membrane-forming strategy, structural continuity, interfacial integrity, thickness, recyclability, and operating stability must be evaluated before powder-level performance can be translated into membrane operation [221].

Adsorption-focused discussion also needs to consider the distinction between material discovery and process relevance. A material may be structurally well designed and have high uptake in optimal conditions, but under humid, mixed-gas, or cyclic conditions it may perform poorly. Accordingly, materials should not be ranked solely by uptake capacity; a more relevant question is whether pore or host–guest interaction descriptors explain selectivity [222] and whether those relationships persist under realistic conditions [223,224,225]. Boundary examples of porous crystalline materials, which go beyond conventional metal–carboxylate frameworks, can be helpful only if they raise the bar for evidence rather than expand the manuscript into a generic review on porous materials [226,227,228].

#### 5.2.4. Evidence-Chain Evaluation for Catalysis, Adsorption, and Small-Molecule Transformations

Evidence for catalysis, adsorption/separation, and small-molecule transformations should be based on evidence chains, rather than individual performance metrics. The evidence chain for catalysis would be structural features → accessible active environment → reaction → mechanism or control evidence → post-reaction stability. In the case of adsorption and separations, the evidence chain would be pore/interaction descriptor → uptake/selectivity trend → mixture/competitive evidence → regeneration and stability. This evidence approach allows us to avoid overgeneralization since a descriptor that is relevant to adsorption may not be relevant for catalysis and vice versa, whereas a structural metal site in one reaction might be an active site in another.

Within the Descriptor-to-Function Evidence Ladder, studies that co-report structural characterization, descriptor analysis, and performance testing but lack a controlled comparison generally remain at Level 2. Controlled comparative series reach Level 3 when structural variation is systematically linked to performance under matched conditions. Level 4 adds function-specific controls and mechanistic or process-relevant evidence appropriate to the claim, such as kinetic and heterogeneity tests for catalysis or competitive adsorption, breakthrough, regeneration, and stability tests for separation. Level 5 requires a prospective descriptor-based prediction followed by experimental validation.

Thus, catalysis, adsorption, and small-molecule transformations reinforce the central point of this review: metal–carboxylate connections, ligand functionality, pore environment, and supramolecular interactions are candidate design variables rather than independently validated explanations of function. These features become transferable design principles only when linked to function through controlled comparisons, function-specific validation, and stability evidence. Representative cases are evaluated according to their descriptor basis and evidentiary roles in Table 5.

### 5.3. Bioactivity and Biomedical Relevance

Bioactive coordination compounds require more stringent validation of their descriptors against functions than other coordination complexes [232,233,234]. The bioactive species in question may not be identical to the crystallized compound, since in the medium it can undergo solvation effects, hydrolysis, ligand substitution, redox transformation, protein interactions, aggregation, metal release, or partial dissolution. Thus, crystal structures, descriptor outputs, and docking poses or scores can support hypotheses about biological activity, but they do not constitute direct evidence of such activity.

Carboxylate complexes have been investigated for antibacterial activity and cytotoxic/anticancer or DNA/ROS-related behavior [235,236]. MOF and nano-MOF systems have also been developed for drug delivery [237], imaging, antibacterial disinfection, and cancer therapy [238,239,240,241]. Broad biomedical reviews provide classification and two-dimensional-MOF context [242,243,244]. However, the bioactivities of the metal complexes may depend on the type of metal ion, coordination geometry, ligand field strength, nuclearity, charge, lipophilicity, supramolecular packing, and electronic properties of the complex. Therefore, evaluating bioactivity requires more than structural information alone. For cytotoxicity studies, dose–response data and, as appropriate, normal-cell comparisons, selectivity analysis, medium-stability evidence, and suitable positive controls are important for interpreting activity [245].

In terms of the Descriptor-to-Function Evidence Ladder, many bioactivity investigations remain at Level 2, including crystal structures, method-derived descriptors, docking outputs, and biological assays without causal validation. The comparative series of compounds may achieve Level 3 when the changes in metal center, ligand scaffold, nuclearity, auxiliary ligands, or charges correlate with biological response under identical conditions. Level 4 adds function-specific controls and mechanism-supporting cellular or biochemical evidence. Level 5 uses descriptors prospectively to predict biological selectivity, uptake, or another defined response and then tests that prediction experimentally.

For cytotoxic and antitumor investigations [246], activity screening must be distinguished from mechanism studies [247]. The cited Co(II), Mn(II), and homo- or heteropolynuclear carboxylate complexes combine crystal-structure analysis with descriptor-generating methods such as molecular DFT or Hirshfeld surface analysis; the reported outputs include orbital, electrostatic, charge, or contact descriptors. Some studies also include docking poses/scores, cytotoxicity, or DNA-binding assays [247,248,249,250]. These components are valuable but not interchangeable: molecular DFT calculations and Hirshfeld surface analysis generate outputs that may serve as descriptors, molecular docking generates a binding hypothesis, and a biological assay measures a response. A low IC_50_ value can reflect uptake, lipophilicity, nonspecific toxicity, ligand activity, metal release, aggregation, or another process, while docking does not establish intracellular target engagement. The evidence level therefore depends on the specific descriptor–bioactivity link that is actually tested.

Similar considerations apply to antibacterial or multifunctional biological activity claims. Inhibition zones and initial screening results are useful for selecting active molecules, but mechanism-based claims may require, as appropriate, MIC and/or MBC measurements, strain information, concentration dependence, standard controls, time-kill assays, membrane-integrity tests, ROS generation, metal-release assays, and bacterial morphology evaluation. A multifunctional compound with luminescence, adsorption, antibacterial effects [251,252], and cytotoxicity cannot be interpreted using one universal descriptor. Each function needs to be studied separately.

Molecular docking and Hirshfeld surface analysis remain valuable methods when they answer a defined biological question, while docking poses/scores, ESP distributions, frontier-orbital distributions, and Hirshfeld contact outputs can serve as hypothesis-generating descriptors. Together, they may suggest electrostatic complementarity, hydrogen bonds, hydrophobic contacts, possible biomolecular recognition, and electronic reactivity. Nevertheless, they cannot replace experiments on medium stability, uptake, biological pathways, and selectivity. Mechanism-supported examples can be separated into a Co(III) complex with selectivity/target/pathway evidence [253], a tetranuclear Cu(II) DNA-binding/anticancer study [254], and ROS/GSH-depletion chemodynamic systems [255,256,257].

Thus, bioactivity provides a stringent test of the key idea of this review. Structural and electronic descriptors can produce a set of hypotheses about biological effects, but biological function must be validated in the corresponding chemical and cellular environment. Future studies should go beyond reports of “active complexes” and perform comparative and mechanism-based descriptor–bioactivity correlation. The representative examples of the bioactivity-related studies are shown in Table 6.

## 6. Critical Roadmap and Perspectives: From Structure Reporting to Predictive Design

From the discussion in previous sections, it becomes clear that metal carboxylate coordination polymers and complexes show structural complexity, high chemical tunability, and diverse functions due to the flexible binding capabilities of carboxylates, the diverse coordination preferences of metal centers, the availability of discrete and extended structures, and the increasingly widespread use of crystallographic, supramolecular, electronic, adsorption-related, and biological descriptors. However, these characteristics also highlight a crucial problem in the field: new structural and functional features are discovered faster than transferable descriptor–function relationships are established.

Thus, the next step in development of the field cannot be assessed merely by the number of novel compounds, coordination modes, network topology or functional examples. It should instead be measured by how effectively structural features are translated into descriptors, how rigorously descriptors are connected to function, and whether such relationships can eventually support prediction. In this way, the Descriptor-to-Function Evidence Ladder is proposed as an evaluation heuristic for individual claims and as a roadmap for future study design. The objective of this section is to develop a forward-looking research design based on the critical assessment presented throughout the review.

### 6.1. From Structural Novelty to Descriptor-Based Evidence

Novel crystal structure discovery continues to remain one of the most valuable achievements in coordination chemistry. In the case of metal–carboxylate systems, even slight variations in ligand geometry, denticity, metal center, auxiliary ligand, pH, solvent, counterion, or crystallization conditions can produce different discrete structures, chains, layers, frameworks, or MOF-like structures. This structural diversity is highly useful because it increases the range of possible structures that can be used for further functional investigations.

However, structural novelty alone is not equivalent to function. A new metal–carboxylate coordination motif becomes more significant when it helps answer a transferable question: which structural variable influences a given function under defined conditions and at what level of evidence? A coordination mode can influence network propagation; a metal center can change Lewis acidity, redox properties, emission, or biological activity; an auxiliary ligand can modify packing flexibility or electronic structure; and a pore environment can control guest molecule accessibility. All of these are design variables, but none of them is a design rule until its functional role is established through comparative studies.

Therefore, the critical transition is from “structure plus function” to “structure-derived descriptor plus validated function”. A descriptor should be chosen not because it is readily accessible, but because it answers a particular question regarding the function. Hirshfeld surface analysis becomes relevant when the claim concerns supramolecular contacts. Energy-framework analysis becomes relevant when packing stabilization is discussed. ESP maps become relevant when electrostatic interaction is invoked. TD-DFT calculations become relevant when optical transitions are interpreted. GCMC simulations become relevant when guest access and selectivity are evaluated. Docking becomes relevant when biomolecular interactions are proposed. In each case, the selected method, its descriptor output, the functional claim, and the validation strategy must correspond.

### 6.2. Descriptor Standardization and Evidentiary Boundaries

An obvious challenge for descriptor-guided design is insufficient reporting practice. Crystallographic descriptors like coordination mode, metal–ligand bond lengths, coordination geometry, metal–metal distances, ligand dihedral angles, hydrogen-bonding interactions, aromatic stacking distance, dimensionality, pore size, void volume, and solvent-accessible volume need to be reported with consideration for comparing structures that are closely related. For porous materials, crystallographic voids must be distinguished from experimentally measured porosity. For molecular systems, molecular geometry needs to be distinguished from crystal-packing effects.

For postsynthetically functionalized frameworks, the modified material should be compared explicitly with its precursor to verify framework retention and any intended change or preservation of the local coordination environment [260,261,262]. Framework stability [263] should be assessed under the water, chemical, thermal, or mechanical conditions relevant to the claimed function, with the criterion used to establish structural retention stated explicitly [264,265,266]. Defect engineering can alter local coordination, composition, and pore environments; therefore, defect type and concentration should be characterized rather than inferred from bulk porosity alone [267,268,269]. For amorphous, gel-derived, and glassy coordination materials [270], characterization should distinguish retained local or short-range coordination from the degree of long-range order [271,272,273,274].

The same principle applies to computational and supramolecular analysis. Hirshfeld surface analysis is the method; contact fractions and mapped surface features are outputs that require physical interpretation. Energy-framework analysis generates model-dependent pairwise interaction energies and network representations that must not be conflated with contact frequency. DFT model choice and reporting must state whether the system is molecular, cluster-based, embedded, or periodic and must provide the relevant settings; molecular orbital and ESP outputs are not substitutes for periodic DOS/PDOS, band-structure, extended charge-density, adsorption-energy, or defect-energy descriptors when the claim depends on an extended solid [94]. GCMC, docking, and machine-learning studies should likewise report their model assumptions, targets, data construction, validation, uncertainty, and applicability domain as appropriate.

As important as these reporting principles are, the evidentiary boundaries of the descriptors themselves are equally important. A high contact percentage does not mean that a sensing mechanism is present. A red area on the ESP surface does not guarantee a guest-binding site. A small HOMO–LUMO gap does not guarantee a catalyst or a cytotoxic compound. A simulated adsorption site does not substitute for real isotherms or breakthrough data. A docking pose does not ensure intracellular target engagement. These facts do not negate the importance of descriptors; they just show how descriptors should be used scientifically. A good descriptor–function discussion should identify whether the descriptor is descriptive, correlative, mechanism supported, or predictive.

### 6.3. Comparative Structural Series as the Bridge to Transferable Rules

The most practical route from descriptive coordination chemistry to function-oriented design is the construction of comparative structural series. Isolated compounds can demonstrate that a structure exists and that a function is observed, but they rarely establish a transferable relationship. Comparative series allow the field to test whether controlled changes in metal identity, ligand geometry, auxiliary ligand, nuclearity, dimensionality, pore functionality, or supramolecular packing lead to reproducible changes in function.

Such comparisons can be designed at multiple levels. A metal-varied series can test how Zn(II), Cd(II), Cu(II), Co(II), lanthanide, alkaline-earth, or other centers influence emission, catalysis, adsorption, or bioactivity. A ligand-varied series can test the effects of rigidity, flexibility, conjugation, donor-atom distribution, carboxylate position, or mixed O/N donor environments. A mixed-ligand series can isolate the role of N-donor auxiliaries in capping, bridging, packing, or electronic modulation. A dimensionality series can test whether discrete complexes, chains, layers, and frameworks differ because of changes in connectivity, rigidity, accessibility, or pore environment.

Importantly, comparative series should not be judged only by positive trends. Negative, weak, or unexpected results are equally useful when structural variables are well controlled. If increasing ligand rigidity does not enhance emission, the assumed rigidity–emission relationship must be revised. If a high pore volume does not improve separation selectivity, pore chemistry or adsorption enthalpy may be more important than capacity. If a redox-active metal does not improve catalytic performance, substrate access or stability may be limiting. The ability to explain why a descriptor fails is an essential step toward transferable design rules.

### 6.4. Function-Specific Validation as the Route to Higher Evidence Levels

Section 5 shows that descriptor validation must be function specific. In the case of luminescence and sensing, structural or electronic descriptors are meaningful only when supported by claim-appropriate photophysical or sensing evidence, which may include quantum yields, lifetimes, excitation spectra, Stern–Volmer analysis, spectral-overlap assessment, selectivity, reversibility, guest-response evidence, and post-sensing structural characterization. Enhancement or quenching of emission is a functional phenomenon; the mechanistic explanation needs evidence to distinguish rigidity effect, charge transfer, energy transfer, inner filter effect, dynamic or static quenching, guest binding and framework destruction.

In catalysis and small-molecule transformation, the evidence path is quite different. A crystallographically defined metal center is not necessarily the active site under reaction conditions. Depending on the catalyst type and claim, validation may require controls, substrate-scope and kinetic studies, turnover metrics, heterogeneity or hot-filtration tests, leaching studies, recyclability, and post-reaction characterization. Photocatalytic CO_2_ reduction, in particular, requires blank runs, product identification and quantification, carbon-source verification where possible, selectivity analysis, and catalyst-stability evaluation [275]. For MOF-supported metal or nanoparticle catalysts, it is important to distinguish the role of the framework from that of the active species.

For adsorption and separation, pore descriptors should be related to activation, experimentally accessible porosity, isotherms, adsorption enthalpy, selectivity, regeneration capability, competitive adsorption, humidity tolerance, and breakthrough tests where possible. Single-component uptake and simulated adsorption sites are useful as initial evidence, but they cannot by themselves prove process-level separation. For bioactivity, evidence requirements are even stricter since the active species might change in biological media. Normal-cell controls, selectivity index, medium stability, cell uptake, ROS generation, apoptosis or cell-cycle assays, DNA/protein binding, and metal-release analysis should be considered before structural descriptors are connected to biological mechanisms.

Thus, the Descriptor-to-Function Evidence Ladder is not a universal checklist with fixed requirements for all applications. Rather, it is a conceptual framework that links evidentiary strength to claim strength. Level 0 establishes structural identity only; Level 1 adds descriptive descriptors; Level 2 co-reports descriptors and function without controlled comparison; Level 3 establishes controlled descriptor–response trends; Level 4 adds function-specific validation with mechanism-supporting evidence; and Level 5 uses descriptors prospectively and validates the prediction experimentally.

### 6.5. Data-Assisted Screening and Machine-Learning Descriptors

The trend towards a higher level of evidence will increasingly require data-assisted design; however, such an approach should be critically evaluated. Large structural databases, descriptor extraction, machine learning [276], generative models, and inverse design [277] can help detect patterns that cannot easily be derived from case studies [278,279,280]. Such patterns can encode metal type, oxidation state, ionic radius, coordination geometry, ligand denticity, ligand flexibility, distribution of donor atoms, topology, pore size, specific surface area, charge distribution, hydrogen-bonding ability, adsorption energies, electronic features, and functional indicators targeting particular purposes.

Representative research on synthesis includes machine-learning capture of MOF synthesis intuition from successful, partially successful, and failed experiments [281]. A separate application is phase-transition prediction [282]. Data-driven design for wet-flue-gas CO_2_ capture [283] and machine learning of quantum-chemical properties [284] represent two further data-driven tasks. Other task classes include sulfur-adsorption prediction [285], water-stability screening [286], conductive-MOF structure–activity analysis [287], and an MOF recommendation system [288]. A broader review summarizes machine-learning applications in MOFs [289], whereas generative inverse design [290,291] and transferable MOF representations [292] extend these approaches toward candidate generation and transfer learning. These studies focus primarily on MOFs and reticular materials rather than the full range of discrete metal–carboxylate complexes and coordination polymers, but they provide relevant methodological examples of how descriptor-rich structural data can support prospective screening and prediction.

However, it is important to note that machine learning does not automatically solve the descriptor–function problem. Datasets for functions of coordination compounds are usually small, heterogeneous, and skewed towards positive outcomes. There is high variability in experimental conditions, there are very few negative results presented, and functional indicators might not be comparable. Consequently, a model trained on heterogeneous data would replicate underlying bias rather than chemical principles. Therefore, data-assisted design should be used to select candidates and detect descriptor patterns, rather than as a substitute for chemically interpretable validation.

### 6.6. Inverse Design and Closed-Loop Optimization

In the long term, inverse design could become a major objective of descriptor-based coordination chemistry [293]. Rather than synthesizing compounds first and subsequently asking what functions they might exhibit, a prospective workflow would begin by defining the target function, the desired descriptor profile, and the minimum acceptable evidence level [294,295]. Appropriate design variables could then be selected, including the metal center, carboxylate ligand, auxiliary ligand, synthetic conditions, anticipated coordination mode, topology, pore environment, and supramolecular motif. Candidate metal–ligand systems [296] would be prioritized before synthesis, and their predicted structures, descriptors, and functions would subsequently be tested experimentally. Successful prospective prediction followed by experimental confirmation would correspond to Level 5 evidence in the Descriptor-to-Function Evidence Ladder; the preceding stages provide the structural, comparative, mechanistic, and data foundations required to reach that level.

The resulting workflow can be organized as a closed feedback loop. Data-ready structural reporting, including validated structures, phase purity, stability, and complete metadata, provides the initial foundation. Standardized and unit-aware descriptor extraction then enables the construction of hypothesis-driven comparable series in which key variables are controlled. Descriptor–function claims are subsequently evaluated through function-specific benchmarking under matched conditions and strengthened through appropriate controls and mechanistic evidence. Curated and harmonized datasets can then support interpretable models that explicitly consider uncertainty and feature contributions [297,298]. On this basis, predefined functional targets can be used for prospective screening, inverse design, and candidate prioritization. Selected candidate coordination systems are then synthesized, structurally verified, and independently tested, after which both positive and negative results are used to update the model, refine the hypothesis, and guide the next design cycle.

**Figure 5:**
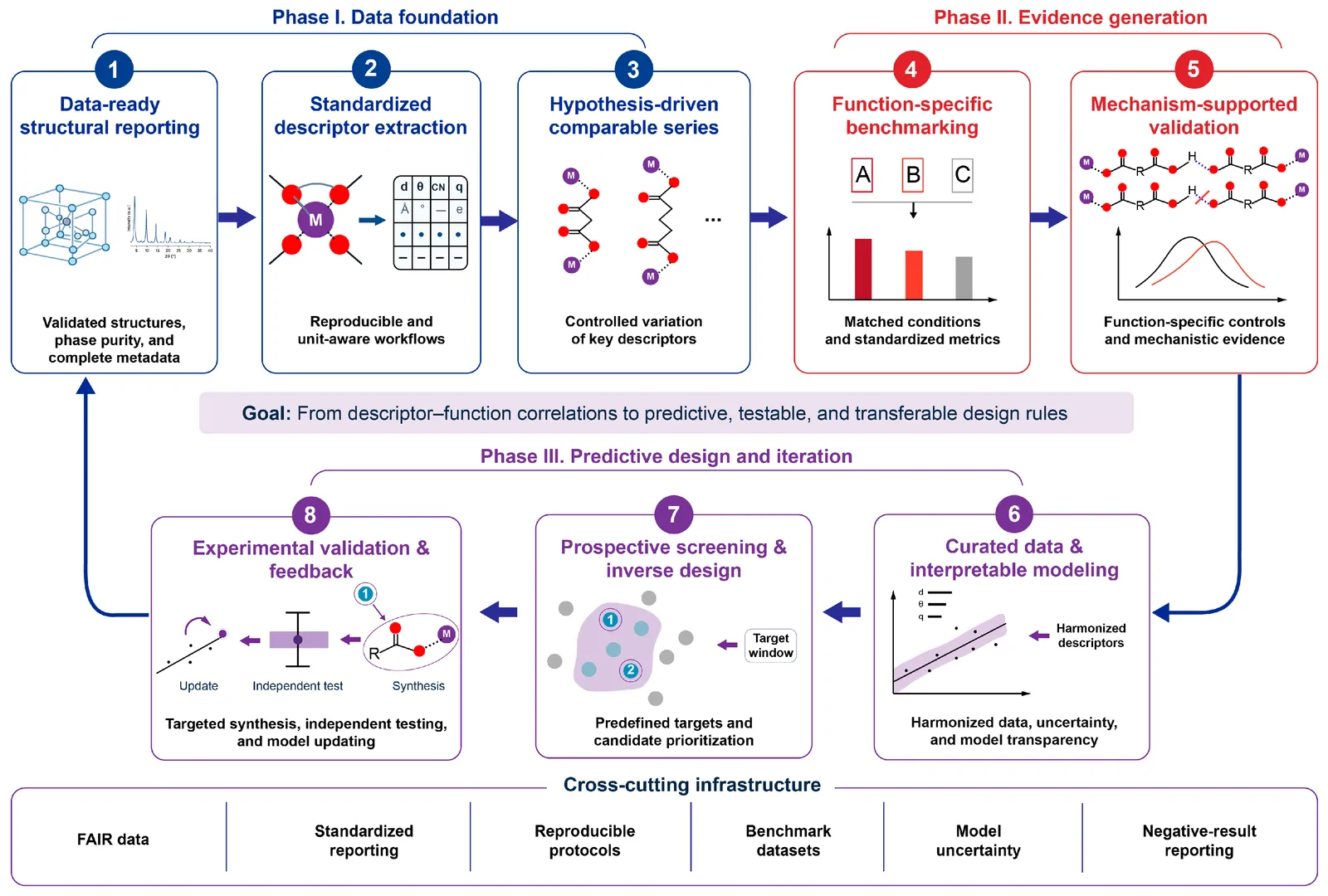
Closed-loop roadmap from data-ready structural reporting to predictive, descriptor-guided function-oriented design. The workflow comprises three interconnected phases: data foundation, including validated structural reporting, standardized descriptor extraction, and hypothesis-driven comparable series; evidence generation through function-specific benchmarking and mechanism-supported validation; and predictive design and iteration through curated data integration, interpretable modeling, prospective screening, inverse design, experimental validation, and feedback-guided model updating. The central goal is to move beyond retrospective descriptor–function correlations toward predictive, testable, and transferable design rules. FAIR data, standardized reporting, reproducible protocols, benchmark datasets, explicit treatment of model uncertainty, and negative-result reporting provide cross-cutting infrastructure for the entire workflow. The shaded envelope in Step 6 conceptually represents model uncertainty rather than a specific statistical confidence interval. Candidate nodes in Step 7 represent candidate metal–ligand coordination-system designs rather than isolated ligands, whereas the local coordination motif in Step 8 schematically represents a prioritized candidate entering targeted synthesis and independent experimental validation. A–C denote comparison systems (Step 4); 1 and 2 denote prioritized candidate coordination systems (Step 7).

This workflow is summarized in Figure 5 and operationalized in Table 7. Figure 5 presents the conceptual closed loop from data foundation and evidence generation to predictive design, experimental validation, and iterative model updating. Table 7 translates this roadmap into practical actions that can increase the evidential strength, reproducibility, and transferability of descriptor–function relationships.

### 6.7. Potential Use and Extension Beyond Carboxylate Systems

The Ladder is proposed as a provisional claim-level heuristic, not a formal reporting guideline or validated peer-review instrument. Within this review, one plausible near-term use is as a planning and self-assessment checklist based on Table 3. Reporting or reviews remain as hypotheses for future evaluation; independent application, inter-rater testing, and broader community consultation would be needed before standardization.

The progression from description to association, controlled comparison, mechanism-supporting evidence, and prospective confirmation may be extensible beyond carboxylates, but this has not been demonstrated. Extension would require ligand-specific descriptors, confounders, and validation thresholds: for example, protonation and coordination mode for phosphonates [299]; the balance between metal coordination and hydrogen bonding for sulfonates [300]; ligand-field and spin-state questions for relevant transition-metal N-donor systems [301]; and sulfur-donor type and metal–sulfur coordination mode [302]. These examples are illustrative. The six-level scaffold is a starting hypothesis whose thresholds may require recalibration, not an invariant scheme already validated across ligand families.

## 7. Conclusions

Carboxylate-ligand-based coordination polymers and metal complexes are a diverse class of crystalline coordination systems. This diversity arises from several factors, including the variety of carboxylate binding modes, the flexible geometry and denticity of organic ligands, the wide coordination chemistry of metal ions, and the presence of additional N/O donors and supramolecular interactions. As a result, discrete complexes, one-dimensional chains, two-dimensional sheets, three-dimensional networks, and MOF-like architectures are possible, and numerous functional studies in luminescence, sensing, catalysis, adsorption, separation, small-molecule transformations, and bioactivity have been conducted.

The next step in this research area is not only to discover structures and functions but to test how defined structural features relate to performance. Single-crystal diffraction, Hirshfeld surface analysis, energy-framework analysis, DFT, TD-DFT, GCMC simulation, and molecular docking are methods or models. Their descriptor outputs—such as coordination geometry, contact fractions, interaction energies, molecular orbital distributions, DOS/PDOS, electrostatic-potential distributions, adsorption energies, occupancy estimates, and docking poses—contribute to structure–function reasoning, but none automatically establishes a descriptor–function relationship. Their evidentiary value depends on hypothesis definition, controlled comparison, function-specific validation, and, where possible, prospective prediction.

The Descriptor-to-Function Evidence Ladder introduced in this review proposes a transparent rubric for the evidentiary strength of individual descriptor–function claims. Its proposed contribution is to combine a cross-domain decision logic with function-specific validation modules while separating claim level from overall paper quality. It is not presented as an externally validated scoring system. Structure determination and descriptive descriptor extraction remain essential foundations. Controlled matched comparisons move a claim from co-reporting to a descriptor–response trend; claim-specific controls and orthogonal measurements provide mechanism-supporting evidence; and a prediction specified before a new experiment, followed by independent confirmation, represents the strongest level.

The present perspective also helps clarify some over-interpretations that are common in the literature. Hirshfeld contact percentages do not by themselves establish a sensing mechanism. ESP maps should not be considered as experimentally confirmed adsorption or biomolecular binding sites. HOMO–LUMO gaps should not be used as a sole criterion in explaining emission behavior, catalytic kinetics, or cytotoxicity of studied systems. GCMC simulations and molecular docking are useful for hypothesis generation, but their predictions require function-specific experimental validation, such as adsorption or breakthrough measurements for separation claims and biochemical or cellular assays for bioactivity claims. The above-mentioned limitations do not undermine the power of the approach to studying coordination chemistry based on the use of descriptors but help to make the conclusions more accurate and design implications more generalizable.

While reviews focused predominantly on nanostructured catalysts, general MOF applications, or certain metal–carboxylate families differ from the present review, this particular review considers carboxylate-based coordination systems as structure-rich materials in the context of the evaluation of how various crystal, supramolecular, electronic, adsorption-related, and biological descriptors support the proposed functions to varying degrees. The difference between these approaches to reviewing coordination chemistry is critical because it allows a clearer distinction between descriptive crystal reporting, retrospective rationalization, structure–function comparisons, mechanism-supported interpretations, and predictive design.

Further advances in coordination chemistry will require changes in the way researchers conduct their studies. More research projects should be designed in such a way that they focus on structural series, descriptor reporting, function verification, and feedback-based optimization. Ligands, metal centers, auxiliary donor ligands, topologies, pore environment, and supramolecular interactions should be chosen not only due to their ability to produce new structures but because they are expected to produce specific descriptor profiles. Data-assisted screening and inverse design can accelerate this process but only if the underlying datasets include chemically meaningful descriptors and experimentally verified performance.

It is worth emphasizing that carboxylate-based coordination chemistry has an opportunity to move from structure-rich science toward more design-based studies. The field does not need fewer crystal structures, descriptors, or functional tests. It needs strong relations between these three types of information. With the combination of structural variety, chemically relevant descriptors, strong evidence, and descriptor-guided function-oriented design, future studies have the potential to turn coordination chemistry of carboxylate ligands into a more predictable area of coordination materials chemistry.

## Figures and Tables

**Table 1:** Representative carboxylate-ligand classes, coordination tendencies, and structural consequences.

Ligand Class	Defining Feature	Coordination Tendency	Common Structural Consequence	Descriptor Relevance
Monocarboxylates	One carboxylate group; substituent-dependent steric and electronic environment	Terminal, chelating, or bridging coordination	Discrete complexes or clusters; occasionally extended structures	Local geometry, nuclearity, and packing
Rigid di- or polycarboxylates	Multiple carboxylate groups on a rigid or aromatic scaffold	Multitopic bridging	1D chains, 2D layers, or 3D networks, including porous frameworks	Connectivity, topology, pore dimensions, and rigidity
Flexible or semi-rigid di- or polycarboxylates	Rotatable spacers or multiple accessible conformations	Conformation-dependent bridging	Polymorphs, flexible coordination polymers, or dynamic networks	Conformational response and predictability limits
Intrinsic mixed O/N-donor carboxylates	Carboxylate and N-donor sites within the same ligand	Chelating or multitopic mixed-donor coordination	Discrete or extended mixed-donor structures	Donor-site distribution, metal selectivity, topology, and electronic distribution
Carboxylates with auxiliary N-donor co-ligands	Separate carboxylate and N-donor ligands	Complementary capping or bridging coordination	Mixed-ligand discrete or extended structures	Comparative control of dimensionality, packing, and function

Note: These outcomes represent common tendencies rather than deterministic predictions. The resulting structure also depends on metal identity, stoichiometry, solvent, pH, counterions, temperature, concentration, and crystallization kinetics. “Intrinsic mixed-donor” denotes O- and N-donor sites within the same ligand, whereas an “auxiliary N-donor co-ligand” is a separate ligand.

**Table 2:** Structural descriptors and method-derived descriptor outputs: physical meaning, evidentiary limits, and claim-appropriate validation.

Descriptor	What It Reports	Appropriate Use	Main Evidentiary Limit	Claim-Appropriate Validation
Coordination number and geometry	Local coordination environment, bond lengths, angles, and polyhedral geometry	Comparing how metal centers organize ligands	Does not independently explain catalytic, photophysical, or biological function	Matched analogues and, where relevant, speciation, stability, or post-function characterization
Connectivity and dimensionality	Propagation into discrete, 1D, 2D, or 3D structures	Assessing rigidity, accessibility, and network propagation	Higher dimensionality does not imply better performance	Phase-pure matched series tested under identical conditions
Hydrogen-bonding and aromatic-stacking motifs	Geometrically defined noncovalent packing motifs	Developing hypotheses about packing, rigidity, or recognition	Geometric occurrence alone does not establish energetic or functional importance	Crystallographic criteria, comparative structures, and energy or functional measurements as relevant
Hirshfeld-surface outputs (from Hirshfeld surface analysis): *d*_norm_, fingerprint contact fractions, shape index, and curvedness [81,82]	Surface proximity, relative contact contributions, and surface topology	Comparing intermolecular-contact and packing patterns	Contact fractions and mapped surface features do not measure interaction energy or establish a functional mechanism [109,112,115]	Matched structures; crystallographic geometry; energy calculations when energetic claims are made
Pairwise interaction-energy components and energy-network topology (from energy-framework analysis) [86]	Model-dependent electrostatic, polarization, dispersion, repulsion, and total interaction energies between defined crystal units	Identifying dominant calculated packing interactions	Calculated lattice interactions do not establish functional causality	Full computational settings, related structural series, and relevant experiments
Molecular electrostatic-potential output (from a stated molecular or cluster electronic-structure model)	Model-dependent electrostatic regions	Identifying candidate interaction sites	Electrostatic regions are not experimentally confirmed binding, adsorption, active, or biological sites [109,112]	Binding, guest-response, adsorption, or reactivity measurements appropriate to the claim
Molecular/cluster frontier-orbital outputs: orbital energies, gaps, and localization	Energy separation and spatial distribution of calculated frontier orbitals	Comparing electronic trends and candidate donor/acceptor character	A smaller gap does not imply higher catalytic activity, luminescence, or bioactivity; orbital localization does not assign transitions [69,109,115]	Consistent calculations with spectroscopy, electrochemistry, or TD-DFT as appropriate
Periodic electronic-structure outputs: band structure, DOS/PDOS, periodic charge density, adsorption energies, and defect formation energies [94]	Extended electronic states, orbital contributions, lattice-wide charge redistribution, and model-dependent energetic quantities	Coordination polymers, MOFs, and other periodic solids when the claim depends on extended connectivity, adsorption, or lattice defects	A periodic ground-state model does not represent solution speciation; results depend on structure, functional, dispersion, spin/U treatment, cell, and defect/loading model	Converged periodic protocol; experimental spectroscopy, adsorption, transport, stability, or defect characterization appropriate to the claim
Excited-state energies, oscillator strengths, and transition character (from TD-DFT)	Calculated excited states, transition energies, and oscillator strengths	Supporting absorption or charge-transfer assignments	Does not fully explain solid-state emission, defects, aggregation, or nonradiative dynamics	UV–vis absorption, excitation/emission spectra, lifetimes, and packing analysis as relevant
GCMC-derived adsorption outputs: uptake, occupancy, binding-site distribution, and selectivity [94,104]	Equilibrium adsorption behavior in a specified structural model	Predicting uptake, occupancy, and selectivity trends	Simulated uptake or sites do not establish activated-material or process performance [116]	Experimental isotherms and, as required, mixture, breakthrough, regeneration, humidity, and stability tests
Docking-derived poses and scores for a specified biomolecular target	Predicted pose and score for a specified biomolecular target	Generating hypotheses about possible recognition modes	A pose or score does not prove target engagement, intracellular activity, or therapeutic mechanism [109,112]	Target-specific binding or biochemical assays, together with speciation, stability, uptake, or pathway evidence as claimed
ML-derived features, predictions, uncertainty, and applicability-domain outputs [105,106]	Learned structure–property correlations and predictions	Screening, prioritization, and prospective prediction	Correlation does not establish causality, synthesizability, or validity outside the training domain	Curated data, robust splits, external validation, uncertainty and applicability-domain reporting, and prospective experiments

Note: A method or model generates descriptor outputs; it is not itself a descriptor. *d*_norm_, normalized contact distance; DOS/PDOS, density/projected density of states; ESP, electrostatic potential; HOMO and LUMO, highest occupied and lowest unoccupied molecular orbitals, respectively; TD-DFT, time-dependent density functional theory; GCMC, grand canonical Monte Carlo; ML, machine learning. The cited cases illustrate evidentiary boundaries rather than the overall quality of the papers. Validation examples are claim dependent and do not constitute a universal checklist.

**Table 3:** Operational decision rules for claim-level assignment in the Descriptor-to-Function Evidence Ladder.

Level	Minimum Evidence	What Does Not Suffice	Assignment Rule	Label
0	Validated structure/material; no descriptor–function claim	Characterization alone	Assign when no descriptor-linked function is tested	Structure only
1	A structural or method-derived descriptor is reported	A calculation or map without a linked functional test	Assign when no descriptor-based functional hypothesis is tested	Descriptive
2	Descriptor and response measured for the same system	Multiple assays on one compound or a post hoc explanation	Assign when no matched variation or discriminating intervention is available	Co-reporting
3	Matched series/intervention varies a descriptor-related variable; function is measured under matched conditions	Unrelated compounds, unmatched tests, or several uncontrolled changes	Assign when descriptor variation tracks a reproducible response with major design variables controlled	Controlled trend
4	Level 3 plus claim-specific controls and orthogonal evidence that discriminate alternatives	One generic control, docking alone, or a calculated restatement	Assign when function-specific evidence supports the mechanism; avoid ‘proved’ unless alternatives are excluded	Mechanism supported
5	Prospective descriptor-based prediction confirmed by an independent experiment	Retrospective fitting or validation on derivation data	Assign only when prediction precedes the new experiment	Prospective confirmation

Note: Levels apply to individual descriptor–function claims, not paper quality. If a required criterion is missing or ambiguous, assign the lower level and state why. Predictive and mechanistic claims are assessed separately. These author-proposed rules reduce but do not eliminate judgment and require inter-rater testing.

**Table 4:** Representative luminescence and sensing studies evaluated using the Descriptor-to-Function Evidence Ladder.

Representative System	Function or Analyte	Descriptor Basis	Evidence Level	Critical Evidence-Chain Assessment	Key Reference
Cd(II) 4-acetylphenoxyacetate coordination polymer	Solid-state photoluminescence	SCXRD descriptors, Hirshfeld-surface contact outputs, and emission data	Level 2	Structure and emission are co-reported, but no controlled descriptor–response series is established	[128]
Dinuclear Cd(II) 2-formylphenoxyacetate/1,10-phenanthroline complex	Fluorescence	SCXRD, aromatic contacts, and fluorescence data	Level 2	Single-system, mainly post hoc structure–emission interpretation	[129]
3D Cd-MOF chemosensor	Fe^3+^, Cr(VI) oxyanions, TNP, and colchicine	Framework descriptors, titration, Stern–Volmer analysis, and quenching interpretation	Level 2	Function-specific measurements are reported, but no controlled structural series establishes a descriptor–response trend	[145]
Related Zn(II)/Cd(II) (d^10^) MOFs	Fe^3+^/Cu^2+^ and TNP sensing	Metal-varied but non-isostructural structures and sensing measurements	Level 2	Metal substitution coincides with changes in coordination and topology, so the pair is not a matched Level 3 series	[123]
3D Co(II) coordination polymer	Ion and TNP sensing; CO_2_ adsorption; antibacterial activity	One framework combined with several functional tests	Level 2	Each unrelated function requires its own descriptor and function-specific validation	[155]
EuBDC and TbBDC lanthanoid MOFs	Ag(I), Fe(III), Cr(III), and Cr(VI) sensing	Lanthanoid comparison, emission analysis, nonlinear Stern–Volmer behavior, and water/pH stability	Level 3	A related lanthanoid series supports a comparative sensing trend; nonlinear Stern–Volmer analysis and stability alone do not meet the orthogonal-evidence requirement for Level 4	[156]
Cyclodextrin-based Pb(II) metal–organic nanotube	Turn-on H_2_S detection in living cells	Selectivity, spectroscopic interaction evidence, and cell imaging	Level 4	Methodological boundary example; it is not direct evidence for carboxylate-ligand systems	[152]

Note: Evidence levels refer to the specific descriptor–function claim stated in each row and do not rank emission intensity, detection limit, sensing performance, or the overall quality of the paper. The lower level is retained when a prerequisite for the next level is not documented. The cyclodextrin-based nanotube is retained solely as an out-of-scope methodological comparator. SCXRD, single-crystal X-ray diffraction; BDC, benzene-1,4-dicarboxylate; TNP, 2,4,6-trinitrophenol.

**Table 5:** Representative catalysis, adsorption/separation, and small-molecule-transformation cases evaluated through descriptor–function evidence chains.

Function	Representative System	Descriptor Basis	Evidence-Chain Assessment	Claim-Appropriate Validation	Key Reference(s)
Photocatalytic CO_2_ reduction	Ca(II), Cu(II), and dinuclear Gd(III) carboxylate complexes	SCXRD descriptors, Hirshfeld-surface/DFT-derived outputs, and product formation	Primarily Level 2; no controlled descriptor–response series	No-catalyst, dark, and no-CO_2_ controls; calibrated product analysis; carbon-source verification; selectivity and stability	[182,183,184]
Lewis-acid and small-molecule catalysis	Ca(II) and Ba(II) coordination polymers based on the same ligand platform	Metal-node and structural descriptors, DFT-derived outputs, and catalytic tests	Potential comparative evidence only when tested under matched conditions	Blank, ligand, and metal-salt controls; kinetics; TON/TOF; substrate scope; leaching and post-reaction characterization	[59,60]
Low-dimensional complex catalysis	Cd(II) and Gd(III) carboxylate complexes	Nuclearity and packing descriptors, DFT-derived outputs, and catalytic tests	Primarily Level 2; packing or electronic descriptors do not identify the catalytically active species	Catalytic-origin, heterogeneity, leaching, and stability tests as applicable	[69,115]
Framework-node catalysis	Cd(II) dicarboxylate coordination polymer and representative MOF-catalysis concepts	Cd–carboxylate environment, chain architecture, and site accessibility	Catalytic activity is reported, but the crystallographic Cd site is not established as the active species	Substrate access, hot filtration, leaching, recycling, and post-reaction characterization	[158,171,229]
Supported or immobilized catalysis	Au–Pt/UiO-67 and Pt–Co/MIL-100(Fe)	Nanoparticle dispersion and framework support environment	The framework mainly serves as a support rather than the intrinsic active species	Hot filtration, leaching, metal speciation, reuse, and post-reaction characterization	[193,194]
CO_2_ adsorption and stability	Mg-MOF-74/MCF composite and broader adsorption frameworks	Open metal sites, support environment, pore accessibility, and adsorption enthalpy	Relates stability and uptake, but process relevance needs operating-condition evidence	Cycling, humidity, mixture adsorption, or breakthrough as required by the intended use	[200,201,202]
Light-hydrocarbon separation	Porous MOFs and imidazole-functionalized Zn-MOFs	Pore dimensions, functionalized channels, and host–guest interactions	Descriptor-supported selectivity; process relevance depends on mixture evidence	Mixture breakthrough, regeneration, and structural stability	[206,207,230]
Synthesis-condition-controlled separation	Solvent-regulated layered Zn-MOFs	Solvent-dependent architecture and pore environment	Controlled synthesis variables support comparison; single-component adsorption alone is insufficient for process claims	Reproducibility, competitive adsorption, breakthrough, and stability	[212]
Out-of-scope porous-crystal comparator	COF review on uranium adsorption and photoreduction	Pore recognition, charge distribution, adsorption, and photoreduction evidence	Methodological boundary only; not direct evidence for metal–carboxylate systems	Explicitly retain as an out-of-scope comparator	[226]
MOF-derived OER electrocatalysis	Co nanoparticles in N-doped carbon derived from a Co-MOF	Co dispersion, N doping, surface area, and ECSA	The active material is the derived Co/N–C composite, not the intact MOF	OER metrics, durability, and post-reaction characterization	[231]

Note: The table evaluates the stated descriptor–function claim rather than catalytic activity, adsorption capacity, separation performance, or overall paper quality. Level-related wording is conservative: when matched comparisons, function-specific controls, or mechanistic evidence are not documented for the stated relation, the claim remains at the lower supported level. Validation examples are claim dependent rather than universally mandatory. Experimental and reporting controls for photocatalytic CO_2_ reduction should follow claim-appropriate best-practice guidance.

**Table 6:** Representative bioactivity and biomedical studies evaluated through descriptor–bioactivity evidence chains.

Biological Function	Representative System	Descriptor Basis	Evidence-Chain Assessment	Claim-Appropriate Validation	Key Reference(s)
Cytotoxicity screening	Co(II) carboxylate/N-donor complex	Metal identity and coordination descriptors, molecular DFT outputs, and cytotoxicity	Structure and activity are co-reported; causal descriptor–response relation is not established	Normal-cell comparison, medium speciation/stability, uptake, and mechanism-supporting assays as claimed	[247]
Cytotoxicity screening	Mn(II) amino-acid-derived carboxylate complex	Metal/ligand structural descriptors, Hirshfeld-surface outputs, and cytotoxicity	Primarily activity-screening evidence	Dose–response, selectivity, medium stability, and mechanism-supporting assays	[248]
Anticancer and DNA-related activity	Homo- or heteropolynuclear complexes	Nuclearity, DNA-binding data, docking-derived poses/scores, and anticancer assays	Nuclearity is a candidate variable, not an independently validated causal descriptor	Binding validation, uptake, speciation, and cellular mechanism evidence	[249]
Nanoparticle-mediated cytotoxicity	Zn(II) tetrazole–carboxylate coordination networks formulated as nanoparticles	Network structure, particle formulation, and cellular response	Biological response may depend on formulation and available species rather than crystal structure alone	Ligand/complex controls, particle and medium stability, uptake, normal-cell selectivity, and mechanism as claimed	[258]
Drug delivery and enhanced cytotoxicity	Ca(II)–nicotinate nano-bio-coordination polymer loaded with curcumin	Carrier structure, reversible conversion, drug loading, and cellular response	Provides carrier–drug and mechanism-supporting cellular evidence; not a ligand-field example	Carrier/drug controls, loading and release, particle stability, uptake, selectivity, and apoptosis evidence	[259]
Antibacterial activity	3D Co(II) supermolecular coordination polymer	Co(II) coordination and supramolecular framework	Screening evidence; structure does not establish the antibacterial mechanism	MIC/MBC, concentration dependence, strain controls, and mechanism tests	[155]
Mechanism-supported anticancer benchmark	Co(III)–cyclam flufenamato complex	Metal-based activity, selectivity, target/pathway evidence	Stronger function-specific and mechanism-supporting evidence	Selectivity, target engagement, pathway markers, and cellular validation	[253]
DNA- and ROS-related bioactivity	Cu-based complexes	Metal/ligand environment, DNA interaction, ROS, and cellular evidence	Demonstrates function-specific evidence beyond docking alone	DNA/protein interaction, ROS, uptake, speciation, and cellular validation as appropriate	[254,255]
Framework biomedical example	Nanoscale Fe-MOF carrier and imaging platform	Framework composition, particle size, loading, release, and imaging	Illustrates formulation-dependent biomedical evidence	Particle stability, release, uptake, biocompatibility, and in vivo relevance as claimed	[238]

Note: The table evaluates each stated descriptor–bioactivity claim rather than biological potency or overall paper quality. When a paper contains several biological claims, each should be rated separately. Validation items are claim dependent and do not constitute a universal checklist. Cellular activity may depend on dissolution, speciation, particle properties, release, and uptake; crystallographic or calculated descriptors alone do not establish a biological mechanism. IC_50_, half-maximal inhibitory concentration; MIC, minimum inhibitory concentration; MBC, minimum bactericidal concentration; ROS, reactive oxygen species; CP, coordination polymer; MOF, metal–organic framework.

**Table 7:** Action-oriented roadmap from structural reporting to predictive, descriptor-guided function-oriented design in carboxylate-ligand-based coordination systems.

Roadmap Stage	Current Common Practice	Main Limitation	Recommended Action	Evidence Implication
Structure discovery	Report new complexes, coordination polymers, or MOF-like architectures	Structural novelty is disconnected from a functional hypothesis	Predefine the target function and candidate structural variable	Level 0–1; Level 2 only after function is tested
Descriptor selection	Add available descriptors after structure determination	Descriptor choice is method driven rather than hypothesis driven	Select descriptors for a predefined functional question	Level 1; Level 2 after descriptors and function are co-reported
Comparative design	Study isolated or unrelated compounds	No controlled descriptor–response trend can be established	Construct matched series under matched test conditions	Level 3 only when a controlled trend is established
Function-specific validation	Report performance without claim-specific controls	Performance is not linked rigorously to the proposed descriptor	Apply photophysical, catalytic, adsorption, separation, or biological validation appropriate to the claim	Level 4 when function-specific and mechanism-supporting evidence is added
Mechanism-supporting evidence	Explain function using descriptors alone	Plausible alternatives remain untested	Combine controls, spectroscopy, post-function characterization, and claim-relevant mechanistic probes	Supports Level 4; avoid “mechanism confirmation” unless credible alternatives are excluded
Data-assisted screening	Use small or inconsistent datasets	Models may reproduce bias and lack transferability	Curate comparable data, report uncertainty, and include negative or weak results	Enabling step; no Evidence Ladder level by itself
Inverse design	Synthesize first and interpret afterwards	Design remains retrospective	Predefine a target descriptor profile, predict candidates, and test them experimentally	Level 5 only after prospective experimental validation
Closed-loop optimization	Stop after one successful material	Generality and transferability remain unclear	Update models and hypotheses using positive and negative experimental results	Sustains Level 5 when successive predictions are tested prospectively

Note: Roadmap stages do not automatically confer an evidence level. Level 0 establishes structural identity; Level 1 adds descriptive descriptors; Level 2 co-reports descriptors and function without controlled comparison; Level 3 establishes a controlled descriptor–response trend; Level 4 adds function-specific validation with mechanism-supporting evidence; and Level 5 prospectively predicts a defined response and validates that prediction experimentally.

## Data Availability

No new data were created or analyzed in this study. Data sharing is not applicable to this article.

## Abbreviations

The following abbreviations are used in this manuscript:

0D/1D/2D/3D	zero-/one-/two-/three-dimensional
BDC	benzene-1,4-dicarboxylate
COF	covalent organic framework
CP	coordination polymer
DFT	density functional theory
DNA	deoxyribonucleic acid
ESP	electrostatic potential
FAIR	Findable, Accessible, Interoperable, and Reusable
GCMC	grand canonical Monte Carlo
HOMO	highest occupied molecular orbital
IAST	ideal adsorbed solution theory
IC_50_	half-maximal inhibitory concentration
IR	infrared
IRMOF-3-LA	lactic-acid-modified isoreticular metal–organic framework-3
LUMO	lowest unoccupied molecular orbital
MBC	minimum bactericidal concentration
MCF	mesocellular siliceous foam
MEP	molecular electrostatic potential
MIC	minimum inhibitory concentration
ML	machine learning
MOF	metal–organic framework
NBO	natural bond orbital
NLO	nonlinear optical
OER	oxygen evolution reaction
PXRD	powder X-ray diffraction
ROS	reactive oxygen species
SBU	secondary building unit
SCXRD	single-crystal X-ray diffraction
TD-DFT	time-dependent density functional theory
TNP	2,4,6-trinitrophenol
TOF	turnover frequency
TON	turnover number
UV–Vis	ultraviolet–visible spectroscopy
XPS	X-ray photoelectron spectroscopy
